# A simple and predictive phenotypic High Content Imaging assay for *Plasmodium falciparum* mature gametocytes to identify malaria transmission blocking compounds

**DOI:** 10.1038/srep16414

**Published:** 2015-11-10

**Authors:** Leonardo Lucantoni, Francesco Silvestrini, Michele Signore, Giulia Siciliano, Maarten Eldering, Koen J. Dechering, Vicky M. Avery, Pietro Alano

**Affiliations:** 1 Discovery Biology, Eskitis Institute for Drug Discovery, Griffith University, 4111 Nathan, Queensland, Australia; 2 Dipartimento di Malattie Infettive, Parassitarie ed Immunomediate, Istituto Superiore di Sanità, Viale Regina Elena n. 299, 00161 Roma, Italy; 3 Dipartimento di Ematologia, Oncologia e Medicina Molecolare, Istituto Superiore di Sanità, Viale Regina Elena n. 299, 00161 Roma, Italy; 4 TropIQ Health Sciences, Geert Grooteplein 28, huispost 268, 6525 GA Nijmegen, The Netherlands

## Abstract

*Plasmodium falciparum* gametocytes, specifically the mature stages, are the only malaria parasite stage in humans transmissible to the mosquito vector. Anti-malarial drugs capable of killing these forms are considered essential for the eradication of malaria and tools allowing the screening of large compound libraries with high predictive power are needed to identify new candidates. As gametocytes are not a replicative stage it is difficult to apply the same drug screening methods used for asexual stages. Here we propose an assay, based on high content imaging, combining “classic” gametocyte viability readout based on gametocyte counts with a functional viability readout, based on gametocyte activation and the discrimination of the typical gamete spherical morphology. This simple and rapid assay has been miniaturized to a 384-well format using acridine orange staining of wild type *P. falciparum* 3D7A sexual forms, and was validated by screening reference antimalarial drugs and the MMV Malaria Box. The assay demonstrated excellent robustness and ability to identify quality hits with high likelihood of confirmation of transmission reducing activity in subsequent mosquito membrane feeding assays.

Malaria is a disease resulting from infection by the intracellular protozoan parasite *Plasmodium*. It remains the most significant parasitic disease in the world, causing ~200 million clinical cases and up to 750,000 deaths each year^1^. Substantial efforts are being made not only to reduce the number of clinical manifestations and deaths attributed to malaria, but also to achieve eradication of this disease. *Plasmodium falciparum*, responsible for the most severe form of malaria, is transmitted by female *Anopheles* mosquitoes, which inject sporozoites into humans causing an asymptomatic hepatic infection, followed by the intra-erythrocytic parasite proliferation responsible for the symptoms of malaria and fatal complications of the disease such as severe anaemia and cerebral malaria. Within the host red blood cells, parasites undergo several rounds of asexual replication, while a small proportion develops into sexual forms called gametocytes. Male and female *P. falciparum* gametocytes undergo five stages of maturation (stage I to V), which last about 10 days. Mature gametocytes persist in the peripheral circulation for several weeks^2^,^3^, where they can be taken up by a mosquito during a blood meal. Inside the mosquito midgut mature gametocytes are triggered within a few minutes to differentiate into female and male gametes, followed by mating. Fertilization produces a motile ookinete which further develops, eventually leading to the generation of infective sporozoites migrating to the insect salivary glands.

Malaria control is mostly achieved by prevention of mosquito bites (insecticide-treated nets, indoor spraying)^4^, plus prophylactic and drug treatment, presently based on artemisinin-based combination therapy (ACT). However, emerging resistance to artemisinins in the field is a pressing issue^5^,^6^,^7^. In this context, the development of new antimalarial drugs is critical, as the current choice of drugs is limited. In addition to activity against blood stage asexual parasites, inhibition of gametocyte viability, and a cognate block in parasite transmission, is a necessary complementation for an integrated program of antimalarial intervention.

The last four years have seen the emergence of a considerable number of different approaches to determine the impact of compounds on gametocyte development^8^,^9^,^10^,^11^,^12^,^13^,^14^,^15^,^16^,^17^,^18^,^19^,^20^. Some of these assays are focused specifically on stage IV–V gametocytes, with the aim to identify compounds active against the mature sexual stages, the only ones able to survive and further develop in the mosquito blood meal. While many schizonticides, such as chloroquine, retain some efficacy against young gametocytes (stages I, II, and III)^12^, gametocytes at late stages of maturation are less/not sensitive to them^15^,^21^. The insensitive stage V gametocytes remain apparently quiescent but infectious in the peripheral bloodstream for at least three weeks^3^,^22^. These cells are directly responsible for malaria parasite transmission as they are programmed to sense the environmental changes in the transition from human circulation to the mosquito midgut and to readily transform into male and female gametes. The first event in gamete formation, induced by a decrease in temperature and the presence of the gametocyte-activating factor xanthurenic acid (XA), is the rapid morphological transition from the typical crescent shape of mature gametocytes to a round cell^23^, in a process, essential for the progression of gametogenesis^24^, defined as “rounding-up” (Fig. 1).

Current screening assays for compounds active against gametocytes use a variety of detection methods, including measurement of parasite ATP levels^11^, parasite enzymatic activity^18^ or expression of a gene reporter^12^. Additional image based approaches ascertain gametocyte viability with Mitotracker^15^, or measure the formation of female gametes/zygotes using antibodies against the gamete-zygote surface protein Pfs25^14^,^20^ or specifically assess male gametocyte ability to produce flagellated gametes^13^. The major drawbacks in the current assays include the need to use gametocyte cultures highly purified from asexual parasites^18^ or from uninfected red blood cells, or both^11^, long incubation and assay times imposed by the slow decay of the enzymatic or transgenic reporter activity in unhealthy or dead mature gametocytes^18^ or by the slow accumulation of sufficient signal intensity^15^. The existing assays able to quantify female gamete formation, all based on Pfs25 antibody, require high number of parasites, which limits throughput^14^, and/or additional 16h–24h incubation of activated gametocytes/gametes to achieve adequate signal intensities of fluorescent labelled Pfs25 antibodies^13^,^14^,^20^.

In order to address the need for a high throughput assay to measure the viability of the apparently quiescent mature gametocytes we developed a simple and robust phenotypic assay, solely based on the fact that only viable mature stage V gametocytes are able to form gametes. After compound treatment, mature gametocytes are triggered to undergo gametogenesis and multiple imaging readouts are measured within two hours, including both the total gametocyte numbers and the fraction of those, alive, able to “round up”. Importantly, our high-throughput compatible assay is optimized for use with non-transgenic parasites, allowing the screening of mature gametocytes from any laboratory line or field isolate.

## Results

**Figure 1:**
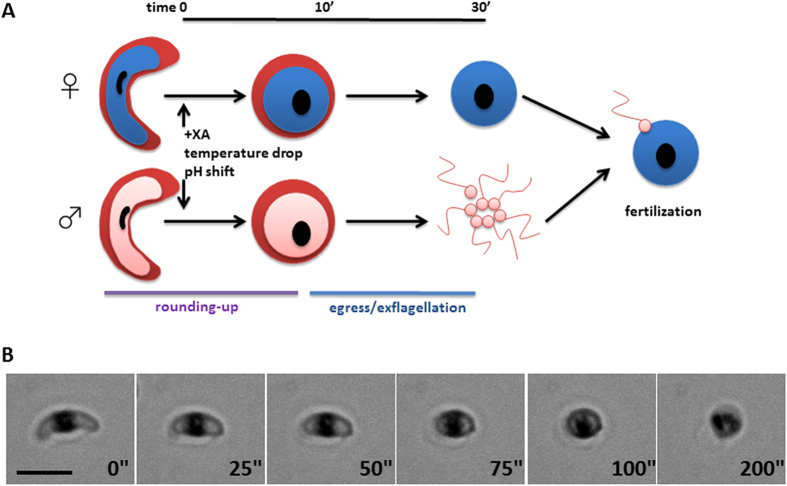
(**A**) The mature gametocyte activation process (modified from^44^). (**B**) Time lapse of gametocyte rounding-up.

Healthy mature gametocytes of *P. falciparum* exposed to a drop in temperature and 40 μM XA are readily triggered to undergo gamete formation. The first step of the process is the fast transition from the typical elongated shape of both male and female gametocytes to a spherical cell, still enclosed in its erythrocyte membrane (Fig. 1). With the only assumption that this process requires the mature gametocyte to be alive, as it needs to sense the change in environment and to respond by modifying its cell shape, we hypothesized that effects of compounds on mature gametocyte viability could be phenotypically assessed from their ability to “round up” and that failure to undergo this developmental step would be predictive of the compounds’ transmission-blocking activity measured in experimental infections of mosquitoes. To this end we devised an imaging-based assay measuring the number of induced spherical activated gametocytes in a format suitable for high throughput screening of large compound libraries, and then compared the data from our assay with published or newly generated data on the transmission-blocking activity in the mosquito of the identified hits.

### Establishing Proof of Principle for the rounding-up assay

**Figure 2:**
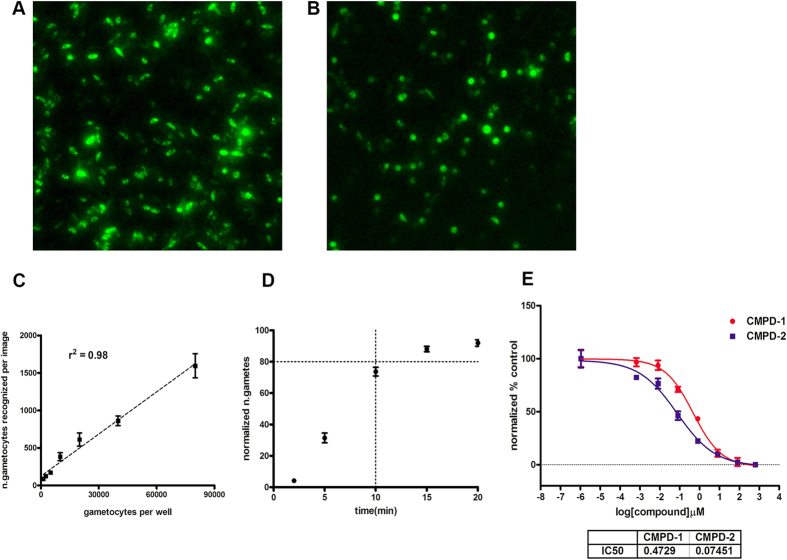
(**A,B**) Representative images of fluorescent gametocytes (**A**) and gametes (**B**) from the parasite line 3D7-PFL1675c:GFP. (**C**) Linearity plot of the number of gametocytes per well determined by microscopy counts or identified by the assay script after Scan^R station automated cytometry imaging. (**D**) Kinetic of rounding-up efficiency in a gamete activation time course with 3D7-PFL1675c:GFP gametocytes. (**E**) Dose-response analysis of the action of CMPD-1 and 2 inhibitors on rounding-up of 3D7-PFL1675c:GFP gametocytes.

To establish the “rounding up” assay, a *P. falciparum* transgenic line expressing a GFP reporter under control of the flanking regions of parasite gene PFL1675c, abundantly transcribed in stage V gametocytes, was initially used. This strain produces elongated mature gametocytes which are readily distinguishable, by fluorescence microscopy, from the spherical rounded up gametes (Fig. 2A,B). Synchronous stage V gametocytes were obtained in 96 wells microtiter plates^18^ to develop a proof-of-principle assay (GFP-GMT) in which GFP detection of elongated gametocytes and of induced spherical gametes was achieved with a Scan^R (Olympus, Germany) modular epifluorescence microscope-based imaging platform. Script parameters were specifically optimized (see Methods for full details) to identify the fluorescent cells and subsequently enable spherical gametes to be distinguished from elongated gametocytes. In this protocol, each fluorescent parasite was identified by calculating a background-corrected fluorescence intensity and an edge segmentation parameter, which defined the object for further analysis as a region of interest (ROI). For each ROI, a circularity factor was then calculated to classify the object as a spherical rounded up gamete or as an elongated, non-activated gametocyte. Further area- and circularity-based gating parameters were incorporated to ignore signal from debris and poorly stained cells, and to specifically identify elongated gametocytes and round gametes, importantly generating image galleries of the individual objects for post-assay quality control inspections.

A linear relationship was observed between the number of gametocytes per well, as determined by manual haemocytometer counts, compared to the automated script estimation. An r^2^ value of 0.98 was obtained and linearity was maintained from 1250 to 80,000 gametocytes per well (Fig. 2C).

In establishing the gametogenesis induction protocol, a time course was performed to define the minimum time necessary to achieve a satisfactory rounding up efficiency before image acquisition. These experiments showed that 10 minutes are sufficient to achieve rounding up of 80% of the induced gametocytes (Fig. 2D).

In order to introduce a reliable baseline control of non-activated gametocytes, we tested two specific inhibitors of the gametocyte rounding up process, Compound 1 and Compound 2 (CMPD-1 and −2). These are potent inhibitors of the *P. falciparum* cGMP-dependent Protein Kinase G (PKG), previously described to block the gametocyte transition from crescent to spherical shape (CMPD-1 IC_50_ = 5.8 nM against recombinant PfPKG)^25^. Mature gametocytes were activated with XA in presence or absence of increasing concentrations of CMPD-1 and CMPD-2; parasites were fixed with 2% paraformaldehyde after 10 minutes and the plate imaged. Results (Fig. 2E) confirmed a higher inhibitory activity of CMPD-2 (IC_50_ = 0.07 μM) compared to CMPD-1 (IC_50_ = 0.47 μM)^25^,^26^, and the former was chosen as the reference rounding-up inhibitor in the assay.

### Assay miniaturization and scale-up for HTS

**Figure 3:**
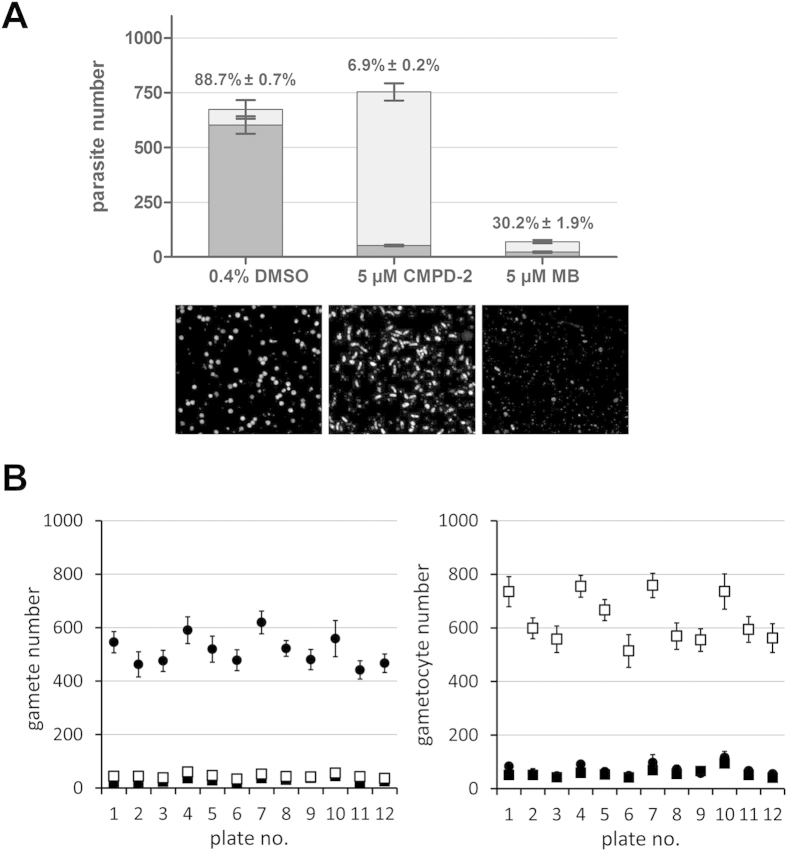
(**A**) Quantitative comparison (n = 17) and exemplar images of positive and negative controls *P. falciparum* 3D7A parasite populations, obtained from the Opera High Content Imaging system. Dark bars = activated gametocyte numbers; light bars = total gametocyte numbers. Error bars represent SEM; percentages above bars indicate % rounding-up. (**B**) AO-GMT assay in-plate control data. Spots identified by the script as gametes (left panel) and gametocytes (right panel) in wells treated with 0.4% DMSO (closed circles); 5 μM methylene blue (close squares) or 5 μM CMPD-2 (open squares). Average numbers ± SEM. Error bars in positive controls are masked by symbols in some cases due to their small size.

**Figure 4:**
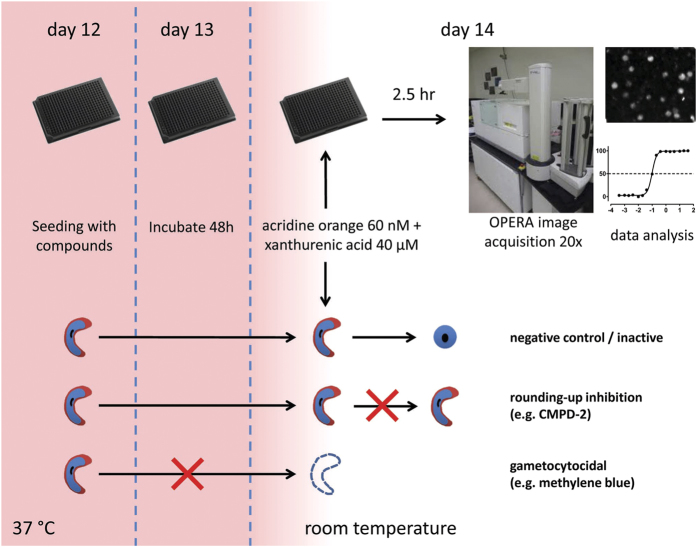
Workflow of the AO-GMT assay and the underlying parasite biology.

To develop an assay not reliant on transgenic gametocytes but potentially applicable to any parasite line or clinical isolate, the assay protocol optimised for proof of principle was modified to introduce a straightforward step for parasite labelling with the fluorescent dye acridine orange (AO). AO has been previously demonstrated to have very low anti-plasmodial activity compared to other fluorescent dyes^27^. The final AO concentration of 60 nM utilized in our assay is 51-fold lower than its reported IC_50_ against 3D7 stage V gametocytes^28^. The introduction of a full-kill positive control treatment, the potent gametocytocidal compound methylene blue (MB)^29^, allowed the assay to simultaneously measure the gametocytocidal and rounding-up inhibitory effects of compounds (Fig. 3A). Importantly, the AO gamete (AO-GMT) assay was adapted to 384 well format for HTS. In all assay runs, the in-plate controls consisted of 5 μM MB and 5 μM CMPD-2 as positive controls (7 wells each) and 0.4% DMSO as the negative control (16 wells). The average ± SEM Z’ values for gamete numbers (CMPD-2 as positive control) and total gametocyte numbers (MB as positive control) were 0.69 ± 0.02 and 0.66 ± 0.02, respectively (n = 12). The DMSO control showed an average %CV of 8.0% ± 0.004% (n = 12). The performance of the assays was extremely stable from the first to last plates with high within-plate reproducibility (Fig. 3B). The final flow chart of the AO-GMT assay is illustrated in Fig. 4.

### Activity of current anti-malarial compounds on mature gametocytes

The AO-GMT imaging assay was used to screen a set of 39 known antimalarial drugs belonging to different chemical classes and acting via different mechanisms on asexual parasites, whose activities have been recently tested against multiple malaria parasite species and stages^30^ and on *P. falciparum* gamete formation^13^,^14^,^31^.

**Figure 5:**
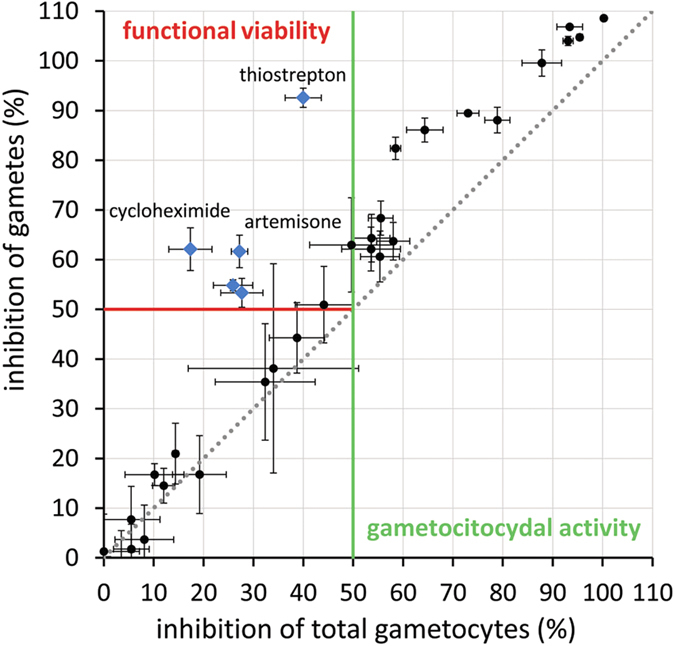
Activity of 39 current and candidate antimalarial drugs on mature *P. falciparum* 3D7A gametocytes and their rounding-up process. Scatterplot of activities of all compounds Compounds were tested at 10 μM concentration. Red and green lines represent 50% activity thresholds for functional viability and total gametocytes readout, respectively. Five compounds showing a higher inhibition on gametes than on total gametocytes are shown as blue squares (thiostrepton, cycloheximide, artemisone, atovaquone and primaquine).

Compounds were tested in dose-response at a maximum concentration of 10 μM for 48 h. In brief, results showed that very few compounds affected gametocyte numbers and viability with an IC_50_ value below 5 μM (Supplementary Table S1 and Figure S1). The low number of active compounds identified is in agreement with previous published assays^13^,^14^,^31^. Analysis of the inhibition readouts at 10 μM showed, as expected, a broad correlation between activity on gametes and on total gametocytes. Generally, active compounds showed a higher activity on gamete inhibition with respect to gametocyte viability (Fig. 5), an observation likely explained by the higher sensitivity of the functional viability readout. Five compounds, namely thiostrepton, cycloheximide, artemisone, atovaquone and primaquine, were found to reduce gamete numbers more than total parasite numbers at this concentration (Supplementary Table S1).

Methylene blue represented the only compound from the panel tested which exhibited strong inhibitory activity against mature gametocytes, with an IC_50_ of 0.17 ± 0.04 μM for gametes and 0.28 ± 0.04 μM for total gametocytes, in agreement with previous reports^13^,^14^,^15^,^20^,^29^,^31^,^32^. Antimalarial drugs belonging to the 4-aminoquinoline, 8-aminoquinoline and antifolate classes have previously been shown to have poor late stage gametocytocidal activity *in vitro*^13^,^14^,^15^,^20^,^32^. As expected, our AO-GMT assay detected activity for most of these compounds only at concentrations above 2.5 μM. Only naphthoquine displayed moderate IC_50_ values in similar ranges for gametes and total sexual forms of 1.14 ± 0.23 and 1.60 ± 0.22 μM. Pyrimethamine, reported to selectively target male gametogenesis^13^,^31^, was inactive in our female gamete assay. Among the aminoalcohols only mefloquine (both racemic and +RS) reduced gamete numbers, with an estimated IC_50_ of ∼5 μM, similar to other recent reports on female gamete assays^13^,^14^,^20^. No specific effect on rounding-up efficiency was observed. Of the seven antibiotic-like compounds tested, only thiostrepton and cycloheximide demonstrated activity against gamete formation. Thiostrepton was the most potent, with an IC_50_ of 1.39 ± 0.31 μM on rounded forms. Interestingly, upon treatment with the two compounds at 10 μM, 92.6% and 62.1% gamete inhibition were observed, respectively, however total gametocyte counts were only slightly reduced, demonstrating that these compounds mainly affected the rounding-up process itself (thiostrepton RND EC_50_ ∼1.97 ± 0.16 μM; Figure S1). Previous work has pointed out that endoperoxides have inhibitory effect on *P. falciparum* sexual stages, although with large assay-related differences^13^,^14^,^15^,^20^,^32^. In our 48 h AO-GMT assay none of the five endoperoxides tested fully inhibited gametes or gametocytes at 10 μM, however artemisone and artesunate reached 50% gamete inhibition at 5 μM.

### Phenotypic Screening of the Medicines for Malaria Ve**n**ture Malaria Box

Medicines for Malaria Venture (MMV) has assembled a “Malaria Box” of 390 compounds with antimalarial activity against asexual blood stage parasites^33^ and made it freely available to the research community for use in the identification of new antimalarial targets and for screening against other parasite stages, as well as against unrelated organisms.

**Figure 6:**
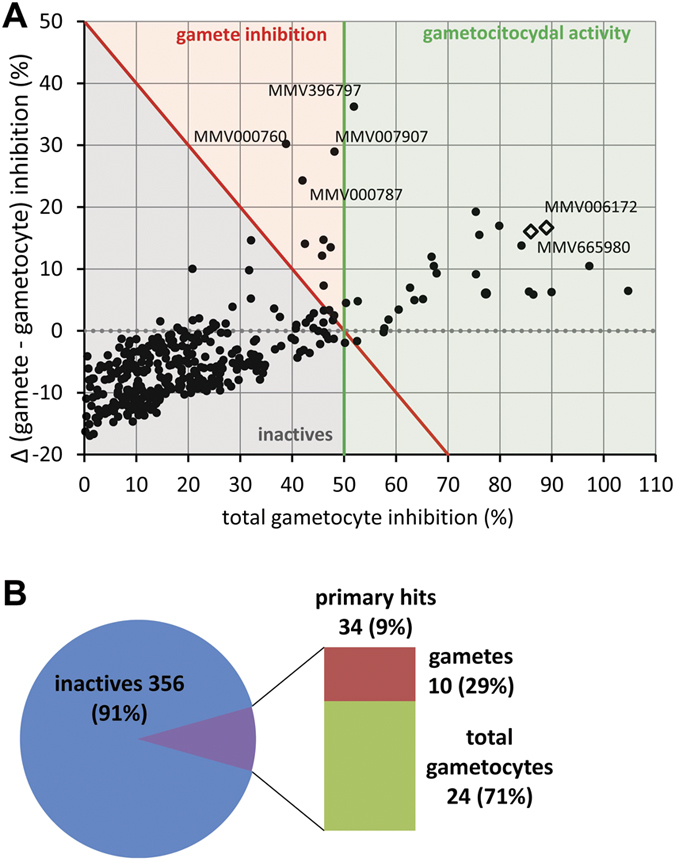
(**A**) Scatterplot of activity on gamete formation and on gametocytes for the entire MMV Malaria Box. Spots are total gametocyte percent inhibition values (X axis) and difference between gamete inhibition and corresponding X value (Y axis) of two biological replicates; red and green lines represent 50% activity thresholds for functional viability and total gametocytes readout, respectively. Dotted gray line indicates gamete and gametocyte inhibition equipotency. White diamonds correspond to the two compounds with the most potent gametocytocidal activity in subsequent dose-response tests. Compounds were screened at 5 μM concentration. (**B**) Overview of screening outcomes; see Supplementary Table S2 for the complete dataset.

Given the amount of transmission blocking data rapidly accumulating on this focused library^34^, the MMV Malaria Box was chosen to further validate the AO-GMT assay. Compounds were screened at 5 μM with a 48 h incubation on stage V gametocytes. A strong linear correlation (r^2^ = 0.957) was observed between the inhibition values obtained by the two assay readouts, namely efficiency of gamete rounding up and total number of sexual forms after compound incubation, suggesting that a compound’s gametocytocidal activity is quantitatively described by either parameter. However, a scatter plot of the values from these readouts shows that with increasing gametocytocidal activity an increasing number of compounds reduced gamete numbers more than total sexual forms (visible as outliers in Fig. 6A). This observation confirms the higher sensitivity of the functional readout over the total count, as already observed with reference antimalarial drugs. The overall comparison of the two assay readouts also indicated that none of the Malaria Box compounds exclusively affected gamete formation without reducing total gametocyte numbers at the same time, i.e. no compound showed an activity similar to that of the CMPD-2 control.

Primary screening identified 37 hits with >50% inhibitory activity on gamete formation. Of these, three compounds were immediately excluded from the active hit list due to autofluorescence or clearly visible artifacts detected upon inspection of the screening images.

The majority of the hits detected at the 5 μM screening dose were gametocyte/gamete inhibitors (24 compounds, 71% of the total hits), while fewer compounds showed a gamete-biased activity (i.e. they decreased the rounding-up efficiency to a higher degree than they reduced total parasite numbers; 10 hits, 29% of the total) (Fig. 6B). Of the gamete-biased hits, MMV000760, MMV000787, MMV007907 and MMV396797 showed gamete vs. total gametocyte inhibitions of 69.0% vs. 38.8%, 66.2% vs. 42.0%, 77.1% vs. 48.1% and 88.1% vs. 51.9%, respectively. These results indicate that such compounds, while killing a proportion of the gametocyte population at 5 μM, also cause sterilization of the remaining, otherwise viable population.

The inhibitory activity of the twenty four anti-gametocyte/gamete hits was confirmed in follow-up dose-response experiments (Table 1), using fresh stock concentrates. The most potent inhibitors of gamete formation were MMV006172 and MMV665980, with submicromolar IC_50_ values of 0.455 ± 0.040 μM and 0.809 ± 0.100 μM, respectively (Figure S1). Twelve more confirmed hits, while still completely inhibiting gamete formation at the highest concentration of 5 μM, showed a lower potency with IC_50_ values in the range of 1.1–2.9 μM. The rest of the confirmed hits only reached inhibition values between 50% and 85% at the highest concentration, and therefore an IC_50_ value was not calculated.

**Figure 7:**
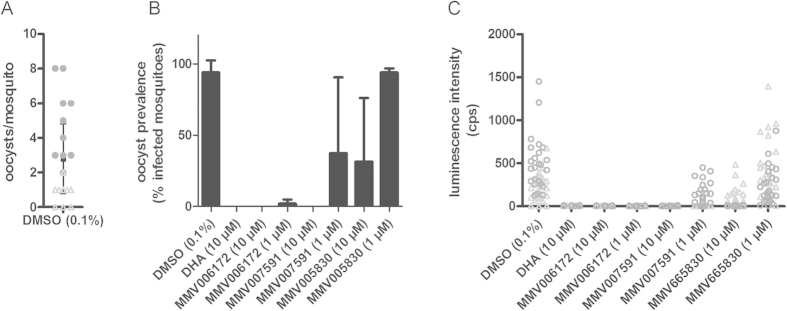
Transmission-reducing activity of three confirmed MMV Malaria Box hits. (**A**) SMFA showed a baseline mean oocyst density of 2.8 mosquitoes per midgut. (**B**) Luminescence-based assessment of oocyst prevalence (% infected mosquitoes). The figure shows average prevalence determined from two independent feeds. Error bars indicate standard deviations. (**C**) Luminescence-based assessment of oocyst intensity. The figure shows luminescence counts in individual mosquitoes from replicate feeds (open circles and triangles). Lines and error bars indicate the average and standard error of the mean.

Five of the 8 confirmed hits with an IC_50_ below an arbitrary threshold of 2.5 μM (MMV665980, MMV019918, MMV000448, MMV665941, MMV667491) have been recently shown to be efficient transmission blocking compounds in dose-response Standard Membrane Feeding Assay (SMFA) experiments (data available at ChEMBL: http://www.ebi.ac.uk/chemblntd). The remaining three compounds (MMV006172, MMV007591 and MMV005830) had never been previously tested by this assay. We therefore assessed the transmission reducing potential of these compounds by SMFA performed on mature gametocytes treated for 24 h at 1 and 10 μM. Microscopic analyses of midguts of mosquitoes in the vehicle control cages showed a baseline infection of 2.8 oocysts on average per mosquito. Analyses of luminescence signals showed that all compounds reduced oocyst intensities by more than 85% at 10 μM (Fig. 7). The most potent hit in our assay, MMV006172, was also the most active in SMFA, resulting in a complete block of infection intensity and prevalence at 10 μM, and near complete block at 1 μM (99,8% inhibition of oocyst intensity and 98% inhibition of oocyst prevalence). Next, MMV007591 completely blocked infection intensity and prevalence at 10 μM and at 1 μM resulted in 80% and 62% reduction of intensity and prevalence, respectively. Finally, MMV005830 was the least effective, resulting in 85% and 69% reduction of intensity and prevalence, respectively, at the highest concentration tested of 10 μM. At 1 μM, no significant reduction was present. In conclusion, these results collectively demonstrate that all the AO-GMT assay confirmed hits tested in SMFA possessed transmission blocking activity in the mosquito. The SMFA and the AO-GMT assay results are therefore in excellent agreement, and provided strong evidence for the predictive power of our phenotypic assay.

## Discussion

This study addressed the need for high throughput screening (HTS) assays to predict the effect of compounds on the mature gametocyte stages of *P. falciparum* to identify molecules able to block the transmission of the parasite from infected humans to mosquitoes. Stage V gametocytes persist in the circulation for several weeks and play an essential role for the continuation of the *P. falciparum* parasite life cycle and thus malaria, making them an attractive drug target. However, the apparently quiescent nature of these parasites has proven to be the main obstacle in the development of assays capable of reliably monitoring their infectivity, thus leaving the SMFA approach as the only functional assay to evaluate compound transmission blocking activity. Although the throughput of this assay has been improved by use of luminescent reporter parasites^35^, its capacity is still too limited for screening of very large compound libraries. Therefore, there is a need for assays with higher throughput that can preselect compounds for subsequent validation in the SMFA.

As the mature gametocyte is highly responsive to environmental changes, ready to suddenly transform into a gamete, we used the first event in gametocyte activation (the “rounding up”) as the most sensitive and fastest phenotypic readout of our assay, which in addition makes it specific to measure the functional viability of the mature gametocytes. Our results indicate that a high level of gametocyte activation is efficiently and reproducibly obtained from highly synchronous, mature stage V gametocytes in 384 well plates.

Solely based on the ability to count and distinguish elongated gametocytes and spherical gametes, our imaging based assay has been validated using reference antimalarial drugs and a pilot screen of a small focused library (390 compounds MMV Malaria Box). The protocol, initially performed on GFP-expressing transgenic parasites, was adapted to assess mature gametocytes of any genetic background by labelling the parasites with the inexpensive AO stain. The imaging protocol and scripts specific for this assay were developed for use on a confocal high-content imaging system, in conjunction with high-throughput liquid handling equipment, to obtain a HTS assay. This approach extends the use of the high content HTS approach recently proposed for asexual^36^ and sexual parasites^15^,^20^ taking advantage of the unique morphology of *P. falciparum* gametocytes and gametes.

In the HTS adaptation of the AO staining assay, the time elapsing between gametocyte activation and automated image acquisition is of the order of 1–2 h. This makes the AO-GCT assay particularly suited to monitor the viability of female gametocytes, as the activated male gametocytes maintain a spherical shape only for about 10 minutes^37^, after which they quickly progress to divide into flagellated microgametes, undetected by our assay. As the sex ratio in *Plasmodium* 3D7A gametocytes is strongly female-biased, with male gametocytes representing about 1/7 of the total gametocytes^38^, the AO-GMT assay is nevertheless reliably monitoring compound activity on the vast majority of the gametocyte population. The assay is however versatile as the protocol can accommodate a cell fixation step after 10 minutes from gamete activation, which, although not practical in HTS of large chemical libraries, enables the imaging of all (male and female) round forms in follow-up experiments on small number of compounds of interest. The comparison of the AO-GMT assay with the two published assays monitoring female gamete formation^13^,^14^,^20^ indicates that our assay does not require expensive detection reagents such as labelled antibodies and/or chemoluminescent immunoassay detection kits, neither the long incubation times after gamete activation (16 to 24 h), necessary for those reagents to achieve satisfactory fluorescence intensity on the gamete surface (Table 2). Importantly, image acquisition within a short time after the induction of gametogenesis ensures that the phenotypic readout truly reflects compound effects on the mature gametocyte, rather than possible confounding effects on female gamete viability. In addition, this is the first assay with the capability to simultaneously count elongated gametocytes and activated gametocytes/female gametes in the same well. Our assay can therefore uniquely distinguish between sterilizing compounds that inhibit gametocyte rounding-up without affecting total sexual forms and gametocytocidal compounds, as well as partial, dose-related effects (Fig. 4). This feature can contribute to the identification of sterilizing compounds with arguably different mechanisms of action compared to gametocytocidal compounds. So far, however, none of the tested antimalarial compounds possessed a female sterilizing activity devoid of inhibition of total gametocytes. Moreover, we found that total gametocyte count is an equivalent predictor of compound activity as gamete formation.

Overall, the protocol strength is the use of a straightforward, rapid and fully automated HTS approach. After a single magnetic purification step on day 4 of gametocytogenesis, ∼40,000 parasites / well are seeded and incubated with compounds in 384-wells imaging plates for 48 h, followed by exposure to XA and AO in a single automated step without washes, and by automated readout acquisition after only 2 h incubation at room temperature. The high-content imaging-based assay has the advantage that the fluorescent microscopy output is a direct measurement of compound effect on individual gametes/gametocytes and not of the total contents of a well, minimizing the interference of background effects. Moreover, the fluorescence signal is specific of parasites, as uninfected erythrocytes lack of nucleic acids reactive to the fluorescent marker. Finally, the possibility to store and review the images provides an important quality control and allows for the quick elimination of false positives/artifacts. At our optimized AO-GMT assay conditions, the current cost of testing a 20,000 compound library is comparable or lower to that of the other gametocytocidal assays available at Griffith, and the current screening capability is ∼25,000 compounds per week.

The screening of the MMV Malaria Box with the AO-GMT assay identified 14 active compounds that showed an IC_50_ < 3 μM. Eight of the most potent compounds were tested in SMFA for validation of their transmission reducing activity, and all of them proved to be active transmission blocking compounds. This confirmation provided solid evidence for the high predictive power of the AO-GMT readout. The two top ranking compounds exhibited submicromolar gametocytocidal IC_50_. The most potent, MMV006172, with IC_50_ = 0.455 μM in our assay, had been previously identified as a late stage gametocytocidal compound with IC_50_ range between 0.420 and 2.6 μM^15^,^16^,^32^,^39^,^40^ . Its transmission blocking activity was confirmed in our SMFA. Interestingly, this compound only caused a partial inhibition at 1 μM in a previous female gamete formation assay^13^, in which however a different assay format and compound exposure time were used. The observation that the longer incubation time in our assay leads to a complete female gametocytocidal effect is in keeping with previous observations that presumed sex-specific effects relate to differences in kinetics rather than an absolute sex preference of gametocytocidal compound activity^14^.

The second most potent hit, MMV665980, (IC_50_ = 0.809 μM) had not been previously identified as a late stage gametocytocidal hit, however it was recently reported to reversibly inhibit (sterilize) male and female mature gametocytes, with IC_50_ = 0.614 μM against female gametes in carry-over format^13^. Also in this case transmission-blocking activity was verified in SMFA (IC_50_ = 1.71 μM). Three more compounds showing gametocytocidal activity below 2 μM in our assay, namely MMV019918, MMV000448 and MMV665941, were previously identified in other gamete^13^ and gametocyte assays^15^,^32^, although with a wide range of potencies, and all confirmed to be transmission-blocking in SMFA. In conclusion, our assay identified and confirmed the activity of all previously reported female gamete inhibitors from the MMV Malaria Box, and all hits from our assay that underwent SMFA validation confirmed their transmission-reducing activity in the mosquito without false positive hits.

Our work showed that it is possible to combine a functional readout on a cell type, the mature *P. falciparum* gametocytes, whose apparently quiescent state makes it an elusive drug target, with a practicable, cheap, fast and fully automated HTS protocol.

Our assay significantly accelerates the possibility to screen very large libraries of compounds to identify quality hits with very high likelihood of showing transmission blocking activity in the gold standard mosquito infectivity assay.

## Methods

### ***P.** falciparum* culture and gametocytogenesis

The strains 3D7A and 3D7-PFL1675c:GFP were cultured *in vitro* as described by Trager and Jensen^41^ with minor modifications. Briefly, parasites were maintained in human type 0-positive RBCs at 5% haematocrit (Hct) in RPMI 1640 medium supplemented with 25 mM HEPES (Sigma), 50 μg/ml hypoxanthine and with the addition of either 10% (v/v) naturally clotted heat-inactivated 0+ human serum (Interstate Blood Bank, Inc.) and 5 nM WR99210 (Jacobus Pharmaceuticals) for 3D7-PFL1675c:GFP, or 5% AB human serum (Sigma) and 2.5 mg/ml Albumax II (Gibco) for 3D7A. The cultures were maintained at 37 °C in a standard gas mixture consisting of 5% O_2_, 5% CO_2_ and 90% N_2_.

At Day−3 of the induction protocol, mid stage trophozoite parasites were isolated on a CS magnetic column (MACS) and VarioMACS separator (Miltenyi Biotec). Fresh RBCs were added to the isolated trophozoites to reach a final parasitemia of 2%, and the hct reduced to 1.25%. After overnight shaking, the Day−2 culture was put under nutritional stress overnight, by keeping the cultures at a high parasitemia of 9% at 2.5% hct and providing only a partial (3/4) exchange of medium. Resulting trophozoite parasites, Day−1, were adjusted to 2–3% parasitaemia and shaken overnight. Gametocyte cultures were maintained in medium supplemented with 50 mM N-acetylglucosamine (NAG; Sigma-Aldrich) in order to clear residual asexual parasites and obtain a virtually pure gametocyte culture. At Day 4, 3D7-PFL1675c:GFP gametocytes were isolated on a CS magnetic column, resuspended in T-25 flasks at 2.5% hct, >50% parasitaemia and cultivated in a hypoxia workstation (Bugbox, Ruskin Intl), until fully mature stage V gametocytes (Day 14). 3D7A gametocytes used in the non-transgenic assay were diluted with fresh blood to a final gametocytemia of 10% and maintained at 2.0–2.5% hct in a standard incubator, with daily change of pre-warmed culture medium until use (stage V; day 12 of gametocytogenesis).

Gametogenesis was measured by treating samples with 50 μM xanthurenic acid (XA; Sigma-Aldrich) in exflagellation buffer (RPMI 1640 with 20 mM HEPES, 4 mM sodium bicarbonate, pH 8.0). After 10 min incubation at room temperature, the numbers of macrogametes and non-activated female gametocytes were determined using Giemsa smears. Specific inhibition of rounding-up was achieved by incubating samples with 5 μM CMPD-2 (kind gift from Dr. D.A. Baker, London School of Hygiene & Tropical Medicine) for 10 min at room temperature^25^. Cultures showing an activation of 95% or above on day 12 were used in the assay.

### Anti-malarial compound stock preparations and handling

A panel of 39 antimalarial compounds (see Table S1 for complete details) was prepared as 10 mM/100% DMSO stock solutions from solids. For IC_50_ determination, DMSO compound stocks were serially diluted in 384-well deep well polypropylene storage plates (Axygen), across the plate. The dilutions made resulted in three concentrations per log dose for each compound tested (14 points, final concentration range 10 μM–0.5 nM). All dilutions were in 4% DMSO, of which 5 μl were stamped into the assay plate, using a Minitrak (PerkinElmer) liquid handler, to a final DMSO concentration of 0.4% v/v. The antimalarials were tested in three biological replicates.

The MMV Malaria Box was received as 10 mM stocks in 100% DMSO. Compounds were reformatted into 384-well format while being pre-diluted twofold into 100% DMSO. DMSO-diluted stocks were stored at −20 °C and once thawed were not repeat freeze-thawed in order to maintain the integrity of the compounds. On the day of assays, stocks were further diluted 25 fold in water and finally 10 fold into culture, to obtain a final screening concentration of 5 μM at 0.4% DMSO. The MMV Malaria Box was screened at a single dose of 5 μM, in two biological replicates. MMV Malaria Box hits were manually cherry-picked, serially diluted as described above and tested in dose-response (14 points, range 10 μM–0.5 nM) in three biological replicates.

### Assay Development: Proof of Principle

As proof of principle, the initial assay was developed using a 96 well plate format with 3D7-PFL1675c:GFP transgenic parasites. Mature gametocyte cultures were plated in 96 wells imaging plates (Ibidi, Germany), in a final volume of 100 μL per well. To test the relationship between the automated gamete count and the initial gametocyte density per well, gametocytes were seeded at different initial densities (1,250–90,000 per well). The rounding-up process was activated 48 hours post-incubation by the addition of 5 μL XA (50 μM final concentration). Gamete numbers were measured in 20 wells using a Scan^R automated imaging cytometry station (Olympus, Japan). Briefly, microplates were imaged with a UPLSAPO 20X N.A. 0.75 (Olympus, Japan) and a filter cube consisting of 470–90 nm excitation, 500nm dichromatic mirror and 520 nm long-pass emission. Gametocytes were seeded at a final average density of 40,000/well, which allowed sampling of a statistically relevant number of events from a single 400 × 400 μm image field from each well. The Scan^R Analysis software (v2.5.1) was used to detect and analyze gametocytes from each well. The ‘edge’ algorithm was used to segment images and shape and intensity thresholds were finely adjusted for each experimental session in order to exclude debris and poorly stained cells. Further area- and circularity-based gating of events allowed complete separation of elongated (gametocytes) versus round (gametes) parasites and compared to manually counted gametes using a haemocytometer. The number of identified parasites per well image (average ± SD) was plotted against the actual number of parasites per well using linear regression.

In a separate experiment, the speed of gametocyte rounding-up was determined by the addition of paraformaldehyde (PFA, 2% final concentration) at different time intervals after stimulation of mature gametocytes with XA and temperature shift.

As candidate positive controls specific for blocking the rounding up process even in presence of XA stimulation, we tested the specific PfPKG inhibitors CMPD-1 and CMPD-2^25^,^26^ in 8 points dose response.

### Acridine Orange Gamete (AO-GMT) assay

For greater versatility, a HTS assay was designed which would allow the use of non-transgenic parasite strains, where gametocytes were stained with a fluorescent dye. For this assay, experimental compounds prepared as described above were dispensed in 384 black clear-bottom imaging plates (Viewplate, PerkinElmer) and added with 20 μl of medium. In each AO-GMT assay plate the following in-plate controls were used: 5 μM CMPD-2 (7 wells); 5 μM Methylene Blue (MB; 7 wells); 0.4% DMSO (14 wells).

Plates were then pre-warmed at 37 °C for a minimum of 30 minutes. 3D7A gametocytes on day 12 of gametocytogenesis from cultures showing acceptable rounding-up upon XA stimulation were seeded in the pre-warmed plates using a Multidrop (Thermo Scientific) 384 reagent dispenser to a final volume of 45 μl and 0.1% hct (∼40,000 gametocytes per well). Plates were sealed with gas-exchange membranes (Breathe Easy, Sigma) and immediately returned to standard incubation conditions (37 °C; 5% O_2_, 5% CO_2_ and 90% N_2_). Extreme care was taken in keeping handling time to a minimum and to ensure pre-warming of all tools and reagents at 37 °C before use.

After 48 hours incubation, plates were retrieved and brought to room temperature. Using a Bravo Automated Liquid Handling Platform (Agilent), half of the culture supernatant volume was slowly aspirated from each well and gently replaced with RPMI supplemented with 25 mM HEPES (Sigma) and 50 μg/ml hypoxanthine, 80 μM XA and 120 nM acridine orange base (AO; Sigma), to final in-well concentrations of XA and AO of 40 μM and 60 nM, respectively. Plates were imaged after 2.5 hours light-protected incubation at room temperature (22.7 ± 0.3 °C). The assay workflow is illustrated in Figure 4.

### Imaging data analysis

Image acquisition and analysis was undertaken on an Opera QEHS micro-plate confocal imaging system (PerkinElmer). Four field images were taken from each well at 5 μm from the bottom of the 384-wells imaging plates using a 20X water immersion objective using 488 nm excitation and 520/35 nm emission, and an exposure time of 280–400 msec.

A custom script based on the spot detection algorithm was developed using the high-volume image data storage and analysis system Columbus 2.5 (PerkinElmer). A first pass selection was used to filter out unwanted spots based on size (accepted range 45–100 px^2^) and background-corrected spot intensity (accepted range between the 5^th^ and 95^th^ percentile of DMSO and CMPD-2 pooled spot populations).

A second pass selection using a spot width-to-length cutoff of 0.65 allowed to discriminate between “spherical” fluorescent gametes, and crescent-shaped fluorescent gametocytes within the selected spot population. With these specifications, it took approximately 20 minutes to screen one full plate.

The use of both a ‘full kill’ (MB) and a ‘no round-up’ (CMPD-2) control in each plate, together with the shape-recognition script allow the simultaneous quantification of compound effects on the overall gametocyte population (irrespective of their ability to round-up, i.e. a “classical” fluorescence-based gametocytocidal assay), as well as to determine the impact on functional viability and efficiency of rounding-up.

Raw imaging data were exported to Excel 2010 (Microsoft) and reduction in gametes and total sexual form numbers, as well as inhibition of rounding-up were calculated as a percentage of the relevant positive and negative controls as below:


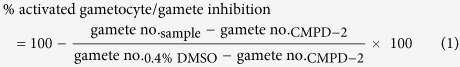



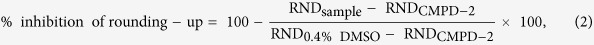


where RND is the proportion of female gametes in each well:


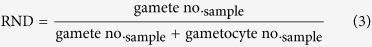



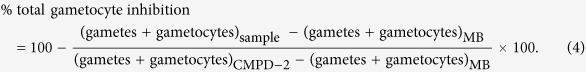


CMDP-2, an inhibitor of rounding up, which completely blocks activation but does not kill gametocytes, was used as either positive control for activated gametocyte numbers and rounding-up (no activation) vs 0.4% DMSO (max activation), as well as negative control for total parasite numbers (no kill and no activation) vs MB (full kill).

The assay quality was evaluated for each readout using the Z’ parameter^42^, defined as:


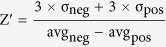


where avg and σ represent the average and standard deviation of the signal obtained from at least 7 wells; and neg and pos represent the relevant negative (maximum signal) and positive (minimum signal) controls used for each readout.

Correlation between number of gametocytes per well determined by microscopy counts or identified by the light microscopy Scan^R assay script were calculated using Excel 2010 (Microsoft). Correlation between total sexual form counts and gamete numbers obtained in the HTS confocal imaging assay was calculated using SPSS v.21 (IBM). Normalized % inhibitions were plotted against log μM concentration of each compound and IC_50_ values were calculated using a variable slope, 4 parameter non-linear regression analysis in GraphPad Prism 5.0. IC_50_ values were not calculated for compounds which did not reach maximal inhibition at the highest concentration tested.

### Standard Membrane Feeding Experiments

Standard Membrane Feeding Experiments were performed as described previously^43^. Briefly, compounds were diluted in DMSO and RPMI1640 medium and combined with mature gametocytes from *P. falciparum* reporter strain NF54-ΔPf47-5’hsp70-GFP::Luc to a final compound concentration of 1 and 10 μM (0.1% DMSO) and incubated for 24 hours. Subsequently, the haematocrit was adjusted to 56% by adding fresh human red blood cells (Sanquin, the Netherlands) and the bloodmeal was fed to 2 day old *Anopheles stephensi* mosquitoes. Eight days post-feeding, ten mosquitoes from each of the two vehicle control (0.1% DMSO) cages were dissected and the baseline oocyst intensity was determined by microscopy following staining of the midguts with 2% mercurochrome. In addition, twenty-four mosquitoes from each cage were homogenized and luciferase reporter activity was determined as described previously^35^.

## Additional Information

**How to cite this article**: Lucantoni, L. *et al*.A simple and predictive phenotypic High Content Imaging assay for *Plasmodium falciparum* mature gametocytes to identify malaria transmission blocking compounds. *Sci. Rep*.**5**, 16414; doi: 10.1038/srep16414 (2015).

## Supplementary Material

Supplementary Information

## Figures and Tables

**Table 1:** Activity of the confirmed AO-GMT hits from the MMV Malaria Box on *P. falciparum* 3D7A gamete formation and on gametocytes.

Compound name	Set	Gamete formation				Total sexual forms				Proportion ofrounding-up	
		IC_50_(μM)‡	±SEM	% inhibition†	±SEM	IC_50_(μM)‡	±SEM	% inhibition†	±SEM	% of 0.4%DMSO control†	±SEM
MMV006172	Probe-like	0,455	0,040	107,33	1,67	0,405	0,090	103,30	0,52	15,12	8,96
MMV665980	Probe-like	0,809	0,100	99,00	2,52			74,82	5,98	16,19	2,75
MMV019918	Drug-like	1,063	0,167*	95,67	2,03	0,825	0,123*	88,47	1,82	44,99	4,34
MMV000448	Probe-like	1,095	0,117*	105,67	1,45	0,643	0,121*	104,08	0,58	30,45	8,09
MMV665941	Probe-like	1,553	0,276*	90,67	1,86	0,843	0,265*	90,84	3,05	74,57	3,22
MMV007591	Probe-like	1,731	0,148	98,33	4,37	1,504	0,269*	98,32	5,68	53,14	10,91
MMV667491	Probe-like	2,354	0,215*	98,67	2,33	1,710	0,427*	93,21	1,03	43,33	10,42
MMV665830	Probe-like	2,469	0,205	87,67	3,84	2,043	0,403*	84,31	2,55	65,52	6,83
MMV396797	Drug-like	2,635	0,113*	93,00	2,65			69,86	5,88	29,36	2,08
MMV000787	Probe-like	2,693	0,172*	86,00	0,58			52,57	1,94	29,09	0,47
MMV019690	Probe-like	2,711	0,124*	93,33	2,60	2,252	0,429*	91,54	1,58	59,44	9,76
MMV006169	Probe-like	2,718	0,271*	88,67	7,26	1,710	0,427*	92,75	7,96	77,61	8,75
MMV019555	Probe-like	2,768	0,176*	89,33	0,33			79,63	1,75	46,61	3,45
MMV666597	Probe-like	2,926	0,269*	95,00	6,56	1,927	0,422*	94,67	4,37	63,97	16,32
MMV000788	Drug-like			80,00	2,52			52,24	1,37	37,14	2,12
MMV396794	Drug-like			80,00	4,16			77,83	1,82	68,81	10,62
MMV006429	Drug-like			75,00	8,62			73,56	4,76	73,16	12,17
MMV665878	Drug-like			74,67	4,91			68,77	2,38	65,04	5,16
MMV007907	Drug-like			74,00	5,13			52,50	3,35	45,41	5,62
MMV000963	Drug-like			73,33	6,01			73,44	5,51	75,53	3,75
MMV000662	Drug-like			71,33	7,54			70,55	6,25	73,43	6,57
MMV396749	Drug-like			57,00	5,13			55,22	2,36	77,27	1,66
MMV665969	Probe-like			56,33	3,48			51,39	1,20	74,19	2,18
MMV306025	Drug-like			50,67	12,33			55,53	9,26	83,65	5,31

^*^IC_50_ value to be considered as approximate (maximal inhibition plateau not reached).

^†^ activity at 5 μM shown.

^‡^IC_50_ values were calculated only for compounds whose inhibition at 5 μM reached at least 85%.

**Table 2:** Comparison of existing assay approaches and parameters with the AO-GMT (confocal microscopy-based, HTS) and GFP-GMT (light microscopy-based, proof of principle) assays.

Assay	AO-GMT assay	GFP-GMT assay	Female assay^14^	Dual M/F assay^13^	Female assay^20^
reagent for readout	acridine orange	GFP	conjugated Mabs	conjugated Mabs	conjugated Mabs
time after compound incubation	2.5 h	10 min	16 h	20 min + 24 h	24 h
plate format	384 wells	384 wells	384 wells	96 wells	384 wells
gametocytes/well	4 × 10^4^	4 × 10^4^	2 × 10^5^	NA	8 × 10^3^
readout(s)	gametocytes and female gametes	spherical gametes	female gametes	male and female gametes	female gametes
Z’	0.69 (gametes) 0.66 (gametocytes)	0.76	0.72	0.43 (male) 0.36 (female)	0.7
