# Supplementary material for: A simple and predictive phenotypic High Content Imaging assay for Plasmodium falciparum mature gametocytes to identify malaria transmission blocking compounds
Source: Sci Rep. 2015 Nov 10;5:16414. doi: 10.1038/srep16414 (PMC4639769; doi:10.1038/srep16414)
Supplement: Supplementary Information [file srep16414-s1.pdf]

A simple and predictive phenotypic High Content Imaging assay for Plasmodium falciparum mature gametocytes to identify malaria transmission blocking compounds

Leonardo Lucantoni, Francesco Silvestrini, Michele Signore, Giulia Siciliano, Maarten Eldering , Koen J Dechering, Vicky M Avery, Pietro Alano

Supplementary Figure S1

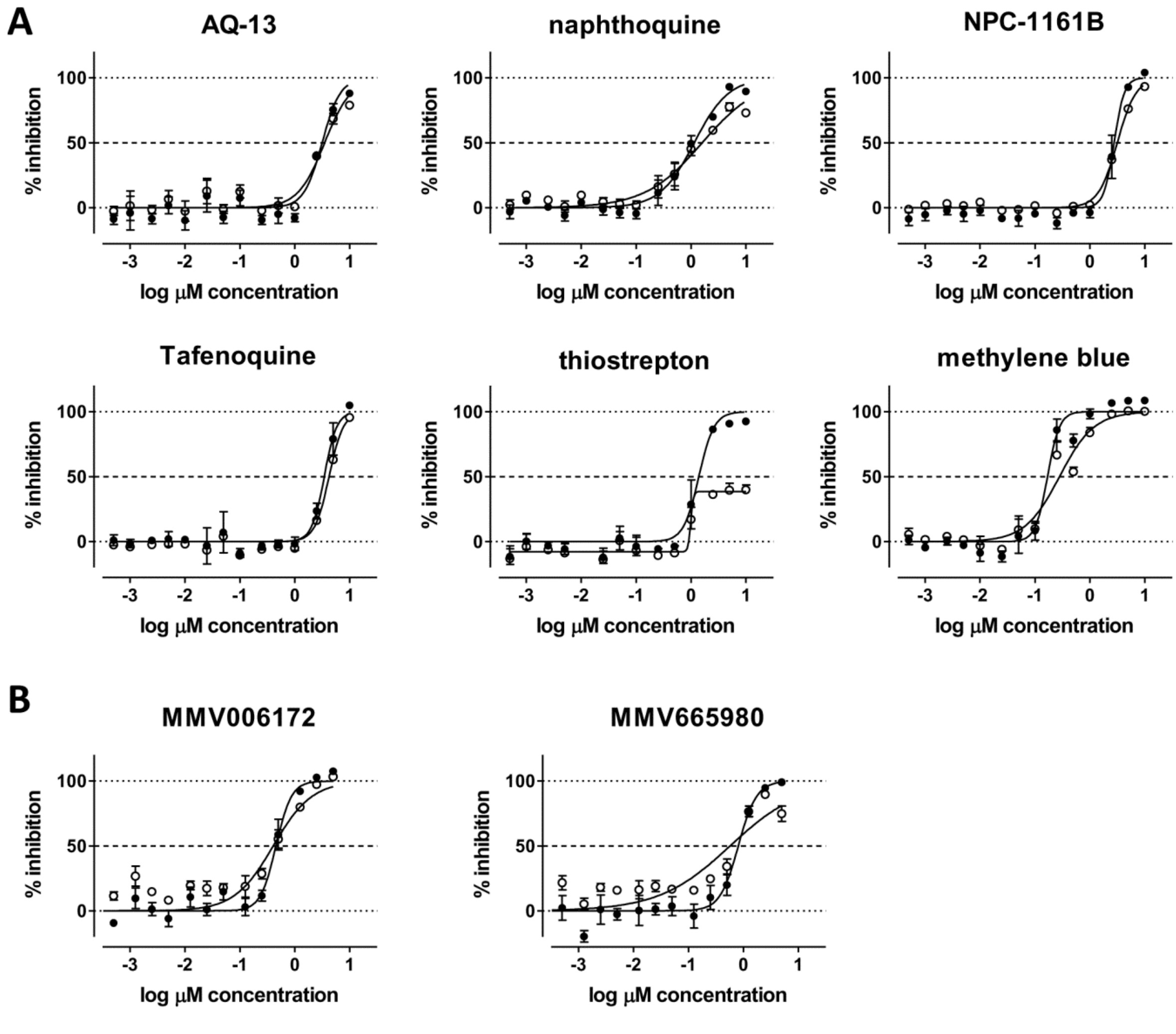

Supplementary Figure S1. Activity of reference antimalarial compounds (A) and hits from the MMV Malaria Box (B) on female gamete formation (filled circles) and total sexual forms numbers (hollow circles). Data are average values and error bars represent SEM (n=3).

Supplementary Table S1

Activity of a panel of antimalarial drugs and compounds on gametes, total sexual forms and rounding up efficiency

		gamete formation				total sexual forms				proportion of rounding-up			
class	compound	IC_{50} (µM)	± SEM	% inhibition†	± SEM	IC_{50} (µM)	± SEM	% inhibition†	± SEM	EC_{50} (µM)	± SEM	% DMSO control†	± SEM
4-aminoquinolines	amodiaquine			62,13	4,40			53,58	5,88			87,03	3,69
4-aminoquinolines	AQ-13	3,15	0,65	88,09	2,59	3,50	0,38 *	78,91	2,52			83,77	1,18
4-aminoquinolines	chloroquine			63,72	3,78			58,03	3,31			92,01	1,03
4-aminoquinolines	hydroxychloroquine			60,62	5,12			55,37	3,92			92,56	2,85
4-aminoquinolines	naphthoquine	1,14	0,23	89,47	0,12	1,60	0,22 *	73,02	2,19			60,12	3,66
4-aminoquinolines	piperaquine			16,73	2,19			10,17	5,88			96,22	0,98
8-aminoquinoline	diethlyprimaquine			20,96	6,12			14,37	0,57			95,32	3,01
8-aminoquinoline	NPC-1161B	2,76	0,34	104,02	0,93	3,15	0,18 *	93,09	1,02			60,28	5,55
8-aminoquinoline	primaquine			53,31	2,92			27,69	4,24			69,46	3,05
8-aminoquinoline	tafenoquine	3,45	0,55	104,77	0,58	4,22	0,38 *	95,42	0,37			71,54	9,15
aminoalcohols	halofantrine			38,11	21,07			34,01	17,10			89,50	5,87
aminoalcohols	lumefantrine			-4,82	1,74			0,82	1,07			99,06	0,52
aminoalcohols	mefloquine (+ RS)	4,75	0,67 *	86,10	2,42			64,35	3,66			52,30	2,52
aminoalcohols	mefloquine (Racemic)	5,11	1,18 *	82,39	2,24			58,49	1,04			52,19	5,68
aminoalcohols	quinine			1,74	5,03			5,54	3,56			98,76	0,58
anilinoacridine	pyronaridine	3,32	0,41 *	106,85	0,42	3,96	0,34 *	93,40	2,58			23,87	3,79
antibiotics	azithromycin			16,75	7,83			19,19	5,39			98,24	2,34
antibiotics	clindamycin			3,65	6,95			8,13	5,87			99,75	0,82
antibiotics	doxycyclin			-2,33	7,82			3,51	6,46			99,68	0,95
antibiotics	fosmidomycin			-3,83	3,73			-5,75	8,28			100,63	0,60
antibiotics	tetracycline			-3,62	3,85			0,82	3,07			99,00	0,59
antibiotics	thiostrepton	1,39	0,31	92,58	1,93			39,98	3,62	1,97	0,16 *	18,55	1,93
antifolates	chlorproguanil	3,08	0,42 *	99,56	2,67	4,16	0,44 *	87,82	3,97			63,25	0,21
antifolates	dapsone			-4,72	3,30			1,46	3,10			99,71	0,48
antifolates	proguanil			50,93	7,71			44,13	5,73			87,23	3,68
antifolates	pyrimethamine			7,72	6,63			5,51	5,75			100,33	0,50
antifolates	trimethoprim			14,51	3,48			12,03	2,24			101,07	0,45
diamidines	pentamidine			68,34	3,44			55,52	2,47			98,27	0,70
endoperoxides	artemether			44,26	7,09			38,73	5,54			99,84	0,45
endoperoxides	artemisinin			35,40	11,73			32,35	10,02			100,47	1,25
endoperoxides	artemisone			61,65	3,28			27,22	1,64			75,03	2,76
endoperoxides	artesunate			62,97	9,48			49,67	8,42			90,27	2,05
endoperoxides	dihydroartemisinin			64,32	4,79			53,68	3,70			93,17	1,53
naphthoquinones	atovaquone			54,82	1,13			25,91	3,95			56,86	1,63
others	cycloheximide			62,10	4,32			17,36	4,34			48,58	3,15
others	methylene blue	0,17	0,04	108,60	0,20	0,28	0,04	100,24	0,20	0,91	0,18 *	76,40	3,78
sulfonamides	sulfadiazine			1,29	7,52			0,03	7,11			81,65	2,22
sulfonamides	sulfadoxine			-1,85	1,72			1,65	2,54			65,65	1,37
sulfonamides	sulfamethoxazole			-4,20	3,38			-6,58	6,01			16,56	2,82

† activity at 10 µM shown
* IC50 value to be considered as approximate (maximal inhibition plateau not reached)

Supplementary Table S2

Activity of the MMV Malaria Box compounds on gametes, total sexual forms and rounding up efficiency. Compounds were screened at 5 µM. Averages of two independent screening replicates ± standard deviation.

ID	Set	gamete formaton inhibition	± SD	total sexual forms inhibition	± SD	% rounding up inhibition	± SD	comments
MMV000448	Probe-like	96,84%	3,52%	79,88%	5,15%	55,40%	6,21%	confirmed hit
MMV000662	Drug-like	68,50%	26,08%	63,55%	20,64%	15,64%	7,34%	confirmed hit
MMV000787	Probe-like	66,29%	29,66%	42,00%	3,93%	45,58%	37,46%	confirmed hit
MMV000788	Drug-like	107,68%	1,29%	97,22%	2,88%	45,11%	13,44%	confirmed hit
MMV000963	Drug-like	60,38%	39,10%	58,56%	31,05%	11,08%	6,94%	confirmed hit
MMV006169	Probe-like	63,94%	21,92%	60,51%	18,47%	5,72%	7,48%	confirmed hit
MMV006172	Probe-like	105,61%	3,19%	88,95%	3,06%	74,51%	17,30%	confirmed hit
MMV006429	Drug-like	77,76%	6,42%	67,29%	2,63%	26,19%	7,95%	confirmed hit
MMV007591	Probe-like	94,61%	3,74%	75,36%	13,87%	53,45%	11,92%	confirmed hit
MMV007907	Drug-like	77,12%	4,48%	48,15%	3,43%	53,66%	9,54%	confirmed hit
MMV019555	Probe-like	60,74%	12,90%	46,02%	9,95%	27,65%	4,56%	confirmed hit
MMV019690	Probe-like	57,40%	25,89%	52,62%	18,50%	13,25%	10,73%	confirmed hit
MMV019918	Drug-like	97,92%	9,66%	84,14%	10,93%	48,28%	9,01%	confirmed hit
MMV306025	Drug-like	83,36%	18,21%	77,31%	15,74%	14,31%	2,75%	confirmed hit
MMV396749	Drug-like	69,66%	13,28%	62,70%	7,96%	18,76%	9,15%	confirmed hit
MMV396794	Drug-like	53,37%	6,23%	46,07%	6,29%	13,88%	3,68%	confirmed hit
MMV396797	Drug-like	88,14%	7,95%	51,91%	3,64%	69,41%	10,16%	confirmed hit
MMV665830	Probe-like	70,34%	8,82%	65,21%	3,74%	13,69%	8,95%	confirmed hit
MMV665878	Drug-like	77,09%	7,80%	67,83%	3,21%	23,46%	9,86%	confirmed hit
MMV665941	Probe-like	91,55%	12,77%	76,05%	18,02%	43,03%	3,52%	confirmed hit
MMV665969	Probe-like	60,91%	2,75%	47,41%	3,17%	24,83%	2,95%	confirmed hit
MMV665980	Probe-like	102,03%	3,86%	85,97%	4,16%	60,30%	7,73%	confirmed hit
MMV666597	Probe-like	91,97%	6,92%	85,62%	6,49%	10,27%	3,04%	confirmed hit
MMV667491	Probe-like	78,82%	6,43%	66,85%	5,22%	27,91%	4,56%	confirmed hit
MMV000248	Drug-like	49,41%	21,77%	46,11%	14,51%	14,06%	7,18%	
MMV000304	Probe-like	44,56%	0,29%	44,10%	1,22%	8,65%	0,97%	
MMV000326	Probe-like	11,22%	0,06%	15,70%	3,31%	0,64%	1,87%	
MMV000340	Probe-like	28,51%	19,64%	33,98%	15,35%	2,85%	4,47%	
MMV000356	Drug-like	3,61%	8,57%	10,03%	14,11%	-1,19%	1,63%	
MMV000442	Probe-like	-3,36%	21,65%	8,76%	18,01%	1,15%	2,78%	
MMV000443	Probe-like	15,50%	2,71%	24,03%	0,40%	1,58%	0,45%	
MMV000444	Probe-like	14,50%	1,03%	16,75%	1,22%	2,97%	5,36%	
MMV000445	Probe-like	45,44%	28,03%	43,41%	19,34%	13,03%	7,96%	
MMV000478	Probe-like	21,02%	2,78%	26,35%	0,21%	4,73%	0,66%	
MMV000483	Drug-like	45,66%	89,01%	46,98%	73,57%	20,50%	26,94%	
MMV000498	Drug-like	3,07%	2,49%	12,77%	4,94%	2,69%	0,41%	
MMV000561	Drug-like	50,81%	46,14%	52,49%	36,84%	4,29%	7,24%	autofluorescent
MMV000563	Drug-like	7,16%	9,74%	8,78%	8,31%	4,30%	6,60%	
MMV000570	Probe-like	14,50%	9,47%	19,36%	10,05%	-0,55%	3,62%	

ID	Set	gamete formaton inhibition	± SD	total sexual forms inhibition	± SD	% rounding up inhibition	± SD	comments
MMV000604	Probe-like	0,72%	9,68%	13,18%	10,36%	-0,22%	0,00%	
MMV000617	Probe-like	-10,05%	18,98%	2,69%	18,13%	1,60%	0,38%	
MMV000619	Probe-like	27,55%	17,42%	34,39%	11,52%	1,34%	1,52%	
MMV000621	Probe-like	28,31%	17,22%	32,08%	11,26%	5,87%	2,34%	
MMV000634	Drug-like	13,82%	12,97%	23,55%	6,43%	0,63%	2,72%	
MMV000642	Probe-like	43,94%	4,76%	42,65%	1,84%	10,28%	1,39%	
MMV000648	Drug-like	39,04%	27,23%	40,52%	21,08%	7,42%	1,63%	
MMV000653	Drug-like	25,06%	28,61%	29,80%	19,50%	5,74%	4,29%	
MMV000699	Probe-like	-1,33%	6,13%	8,23%	5,88%	3,70%	2,15%	
MMV000704	Probe-like	-2,70%	27,41%	9,48%	23,54%	0,83%	1,78%	
MMV000720	Probe-like	22,05%	18,41%	26,77%	9,40%	5,98%	5,90%	
MMV000753	Probe-like	6,38%	20,39%	8,58%	19,53%	3,50%	4,93%	
MMV000760	Drug-like	69,04%	15,66%	38,84%	3,77%	49,19%	17,67%	unconfirmed hit
MMV000839	Drug-like	1,92%	4,38%	13,74%	8,32%	0,23%	2,79%	
MMV000848	Drug-like	25,93%	19,27%	33,82%	12,65%	0,23%	1,84%	
MMV000911	Drug-like	4,71%	2,54%	8,57%	0,07%	2,23%	3,34%	
MMV000917	Probe-like	16,20%	12,30%	20,15%	12,86%	7,15%	0,12%	
MMV000972	Drug-like	47,34%	79,97%	46,02%	70,77%	5,68%	11,28%	
MMV000986	Probe-like	20,50%	0,23%	26,61%	1,08%	3,73%	1,12%	
MMV001038	Drug-like	27,43%	0,80%	32,73%	3,54%	3,22%	1,47%	
MMV001041	Probe-like	24,94%	14,37%	30,33%	11,70%	3,74%	3,44%	
MMV001049	Drug-like	-3,18%	1,54%	10,43%	3,47%	-0,60%	0,43%	
MMV001230	Drug-like	-11,89%	14,63%	2,18%	13,73%	0,61%	1,15%	
MMV001239	Probe-like	-3,78%	5,73%	8,12%	9,57%	1,34%	2,52%	
MMV001241	Probe-like	-9,20%	1,79%	5,57%	6,47%	-0,70%	2,59%	
MMV001246	Drug-like	46,71%	1,71%	32,09%	11,68%	27,34%	13,61%	
MMV001255	Drug-like	12,99%	27,18%	17,60%	19,66%	1,40%	0,07%	
MMV001318	Drug-like	-7,94%	9,04%	2,50%	11,33%	3,72%	0,96%	
MMV001344	Drug-like	19,12%	8,85%	19,16%	7,75%	5,10%	6,07%	
MMV006087	Drug-like	4,46%	4,72%	10,92%	0,91%	-0,68%	0,12%	
MMV006188	Drug-like	23,75%	9,62%	29,23%	8,30%	3,83%	2,57%	
MMV006203	Probe-like	84,54%	27,17%	75,41%	30,44%	28,88%	6,77%	artifact / increased spot fluorescence
MMV006250	Probe-like	7,06%	14,38%	10,40%	16,36%	2,03%	2,05%	
MMV006278	Drug-like	9,87%	23,06%	18,70%	20,02%	2,25%	1,84%	
MMV006303	Probe-like	3,89%	10,67%	9,71%	4,53%	0,39%	0,37%	
MMV006309	Probe-like	12,42%	16,60%	21,79%	13,18%	1,14%	3,60%	
MMV006319	Drug-like	96,19%	19,61%	89,93%	16,22%	18,07%	20,05%	unconfirmed hit
MMV006389	Probe-like	26,01%	18,22%	26,31%	14,74%	4,11%	8,03%	
MMV006427	Drug-like	47,01%	13,99%	44,19%	10,37%	12,64%	1,56%	
MMV006455	Drug-like	23,50%	20,36%	23,62%	17,16%	4,56%	7,53%	
MMV006457	Probe-like	16,98%	26,76%	20,73%	28,79%	-0,74%	1,22%	
MMV006513	Probe-like	44,99%	3,92%	45,01%	2,01%	7,81%	0,15%	
MMV006522	Probe-like	7,42%	3,29%	11,46%	2,14%	1,73%	0,01%	
MMV006545	Drug-like	29,47%	20,40%	33,64%	15,41%	4,90%	0,12%	

ID	Set	gamete formaton inhibition	± SD	total sexual forms inhibition	± SD	% rounding up inhibition	± SD	comments
MMV006558	Probe-like	14,84%	11,80%	17,58%	9,40%	2,22%	7,30%	
MMV006587	Drug-like	-0,05%	3,56%	12,05%	5,42%	0,47%	0,02%	
MMV006656	Probe-like	8,23%	9,49%	11,20%	5,84%	3,07%	2,67%	
MMV006704	Drug-like	-0,69%	13,47%	4,79%	7,91%	1,39%	1,62%	
MMV006706	Drug-like	-14,16%	11,64%	0,95%	12,48%	-0,09%	0,16%	
MMV006753	Probe-like	21,95%	15,24%	23,19%	13,59%	3,20%	5,87%	
MMV006764	Probe-like	24,08%	6,32%	22,08%	7,21%	7,24%	3,74%	
MMV006767	Drug-like	-3,09%	8,89%	9,60%	10,66%	0,30%	0,94%	
MMV006787	Probe-like	23,40%	8,04%	28,60%	10,08%	4,09%	1,82%	
MMV006820	Drug-like	23,08%	22,20%	29,36%	13,77%	3,67%	4,16%	
MMV006825	Probe-like	24,34%	3,04%	24,17%	2,12%	5,11%	4,39%	
MMV006861	Probe-like	22,57%	6,21%	20,88%	4,73%	7,02%	6,72%	
MMV006882	Probe-like	-1,50%	4,16%	5,12%	1,57%	-0,08%	0,42%	
MMV006913	Drug-like	57,39%	67,26%	57,62%	54,81%	10,66%	14,99%	unconfirmed hit
MMV006937	Drug-like	2,25%	11,06%	10,34%	15,65%	-3,04%	1,11%	
MMV006962	Probe-like	2,16%	21,58%	8,08%	14,04%	7,45%	8,00%	
MMV007020	Probe-like	16,24%	8,63%	21,23%	9,21%	5,92%	0,57%	
MMV007041	Probe-like	11,01%	9,77%	13,73%	3,05%	3,17%	1,10%	
MMV007092	Probe-like	12,13%	1,55%	18,71%	7,31%	4,48%	7,87%	
MMV007113	Probe-like	39,88%	3,92%	37,63%	3,58%	12,26%	2,55%	
MMV007116	Drug-like	50,48%	74,13%	47,13%	63,63%	18,00%	27,92%	unconfirmed hit
MMV007127	Probe-like	0,08%	11,62%	12,82%	10,86%	-0,38%	1,30%	
MMV007160	Probe-like	14,93%	0,72%	17,62%	2,77%	2,37%	3,08%	
MMV007181	Probe-like	17,25%	1,52%	25,63%	4,05%	1,26%	0,95%	
MMV007199	Probe-like	8,39%	10,00%	12,13%	5,77%	2,20%	2,10%	
MMV007208	Probe-like	18,37%	3,68%	24,30%	0,99%	4,46%	2,61%	
MMV007224	Probe-like	18,44%	16,90%	17,91%	9,57%	6,78%	2,14%	
MMV007228	Probe-like	2,85%	0,20%	8,13%	4,27%	0,76%	1,05%	
MMV007273	Probe-like	0,01%	10,43%	12,48%	12,05%	-0,17%	1,29%	
MMV007275	Probe-like	1,06%	1,88%	10,13%	0,37%	3,88%	0,72%	
MMV007285	Probe-like	-6,91%	4,10%	-1,02%	3,07%	1,28%	5,68%	
MMV007363	Drug-like	2,10%	10,32%	13,83%	12,79%	0,17%	2,35%	
MMV007374	Drug-like	7,39%	0,51%	16,81%	0,11%	2,20%	3,37%	
MMV007384	Probe-like	41,79%	15,02%	40,82%	14,11%	3,81%	4,01%	
MMV007396	Probe-like	14,30%	20,13%	22,78%	17,08%	1,79%	2,37%	
MMV007430	Drug-like	15,52%	10,00%	16,29%	11,62%	4,37%	2,75%	
MMV007474	Probe-like	12,92%	8,34%	14,75%	8,82%	3,47%	4,32%	
MMV007557	Probe-like	6,09%	2,54%	9,50%	2,33%	2,48%	5,92%	
MMV007564	Drug-like	-6,53%	10,54%	1,35%	0,65%	-0,59%	2,89%	
MMV007571	Drug-like	-10,04%	17,68%	3,03%	16,42%	1,31%	1,01%	
MMV007574	Probe-like	-2,14%	15,28%	11,53%	13,39%	-0,99%	1,78%	
MMV007577	Probe-like	14,50%	15,73%	23,36%	9,87%	1,66%	1,25%	
MMV007617	Drug-like	-0,06%	13,62%	9,65%	13,24%	3,19%	0,88%	
MMV007654	Probe-like	17,29%	16,59%	26,00%	11,83%	1,10%	0,45%	

ID	Set	gamete formaton inhibition	± SD	total sexual forms inhibition	± SD	% rounding up inhibition	± SD	comments
MMV007686	Probe-like	14,46%	1,76%	22,13%	0,90%	2,94%	2,17%	
MMV007695	Probe-like	-14,91%	4,45%	1,78%	8,13%	-1,62%	1,88%	
MMV007764	Probe-like	16,11%	9,73%	16,73%	8,81%	4,65%	5,82%	
MMV007791	Drug-like	19,67%	13,24%	25,42%	10,48%	4,49%	1,87%	
MMV007808	Drug-like	-8,40%	15,62%	4,16%	15,52%	1,50%	0,14%	
MMV007839	Drug-like	39,12%	61,37%	42,23%	50,11%	5,09%	6,55%	
MMV007875	Drug-like	12,28%	19,90%	20,74%	15,37%	2,45%	4,48%	
MMV007881	Drug-like	0,11%	0,87%	7,74%	8,39%	-1,79%	2,57%	
MMV007906	Drug-like	16,31%	19,56%	19,81%	18,77%	0,72%	4,01%	
MMV007977	Drug-like	-3,10%	2,19%	4,70%	4,55%	-1,28%	0,90%	
MMV007978	Drug-like	-3,56%	6,88%	0,57%	2,53%	2,90%	2,32%	
MMV008127	Drug-like	3,95%	3,43%	7,70%	2,09%	2,43%	4,88%	
MMV008138	Drug-like	25,08%	31,21%	30,81%	23,93%	3,88%	0,59%	
MMV008149	Drug-like	10,47%	5,95%	20,51%	9,52%	0,43%	3,17%	
MMV008160	Probe-like	-3,50%	13,42%	2,80%	8,87%	0,76%	2,93%	
MMV008173	Probe-like	-3,70%	7,16%	7,66%	7,92%	2,07%	0,77%	
MMV008212	Drug-like	-2,15%	4,97%	10,01%	6,55%	0,85%	0,14%	
MMV008270	Drug-like	1,72%	1,68%	7,83%	3,70%	-0,06%	3,65%	
MMV008294	Probe-like	-0,68%	3,36%	6,21%	0,62%	-0,50%	2,20%	
MMV008416	Probe-like	16,61%	5,00%	25,10%	4,97%	1,29%	1,78%	
MMV008455	Probe-like	-16,40%	11,97%	-1,37%	12,05%	0,48%	0,71%	
MMV008829	Probe-like	22,46%	13,39%	24,26%	12,47%	2,36%	5,00%	
MMV008956	Drug-like	10,86%	9,28%	14,43%	8,68%	1,64%	5,46%	
MMV009015	Probe-like	7,46%	11,76%	16,62%	12,90%	2,24%	0,93%	
MMV009060	Drug-like	16,99%	1,38%	23,02%	1,03%	4,55%	3,06%	
MMV009063	Drug-like	24,73%	2,03%	28,42%	0,63%	6,18%	4,35%	
MMV009085	Probe-like	16,84%	7,52%	24,53%	7,18%	2,30%	1,65%	
MMV009108	Drug-like	8,71%	7,41%	16,17%	7,90%	4,28%	0,93%	
MMV009127	Probe-like	111,14%	1,83%	104,70%	0,68%			autofluorescent
MMV011099	Drug-like	11,29%	3,14%	16,80%	1,04%	-0,56%	3,75%	
MMV011256	Drug-like	2,20%	0,72%	8,15%	3,84%	0,07%	2,49%	
MMV011259	Drug-like	12,21%	10,46%	15,68%	11,68%	1,47%	3,02%	
MMV011436	Probe-like	-3,28%	1,67%	7,96%	1,15%	2,22%	0,14%	
MMV011438	Probe-like	-2,56%	0,70%	5,10%	4,97%	-1,24%	0,05%	
MMV011522	Probe-like	18,97%	6,25%	19,77%	1,92%	4,17%	10,32%	
MMV011567	Drug-like	7,18%	18,60%	15,61%	20,16%	2,86%	2,72%	
MMV011576	Drug-like	-5,33%	10,76%	8,36%	9,58%	-0,25%	2,09%	
MMV011832	Probe-like	15,71%	1,97%	24,65%	0,66%	0,94%	0,17%	
MMV011895	Probe-like	18,63%	9,26%	21,02%	8,51%	2,16%	5,42%	
MMV011944	Drug-like	19,30%	21,06%	26,15%	18,84%	2,71%	1,13%	
MMV018984	Drug-like	-1,16%	16,22%	10,82%	14,40%	0,80%	1,72%	
MMV019017	Drug-like	17,93%	16,71%	19,12%	12,98%	3,82%	8,51%	
MMV019064	Drug-like	1,51%	13,21%	11,17%	13,38%	2,91%	0,24%	
MMV019066	Drug-like	-7,00%	22,41%	5,28%	20,11%	1,55%	1,23%	

ID	Set	gamete formaton inhibition	± SD	total sexual forms inhibition	± SD	% rounding up inhibition	± SD	comments
MMV019074	Drug-like	-17,64%	6,50%	-1,11%	9,57%	-0,89%	1,47%	
MMV019110	Drug-like	16,98%	3,99%	19,78%	4,89%	1,92%	4,02%	
MMV019124	Drug-like	-1,81%	2,21%	9,87%	2,68%	1,40%	2,21%	
MMV019127	Drug-like	25,12%	14,51%	30,87%	11,30%	3,32%	1,62%	
MMV019199	Probe-like	26,06%	25,46%	24,82%	21,82%	6,13%	7,92%	
MMV019202	Drug-like	1,96%	1,47%	8,34%	3,17%	-0,35%	1,04%	
MMV019241	Probe-like	-0,66%	4,80%	7,18%	0,44%	-1,54%	0,97%	
MMV019258	Drug-like	10,09%	26,87%	18,24%	24,55%	2,82%	0,25%	
MMV019266	Drug-like	40,13%	35,07%	36,54%	30,30%	8,76%	9,71%	
MMV019313	Drug-like	-16,10%	0,31%	0,20%	4,89%	-0,86%	2,41%	
MMV019406	Probe-like	9,40%	19,33%	18,52%	16,92%	2,01%	1,81%	
MMV019662	Drug-like	28,82%	28,76%	32,68%	24,46%	5,20%	2,69%	
MMV019670	Drug-like	33,73%	17,66%	36,72%	10,22%	6,08%	4,97%	
MMV019700	Drug-like	-2,98%	12,34%	7,81%	12,65%	2,49%	0,30%	
MMV019738	Drug-like	2,61%	11,92%	13,98%	11,17%	0,72%	1,30%	
MMV019741	Probe-like	8,76%	14,05%	18,20%	12,78%	1,76%	1,48%	
MMV019746	Drug-like	7,46%	4,54%	15,18%	7,00%	4,16%	0,89%	
MMV019762	Drug-like	11,43%	2,57%	13,15%	6,71%	3,79%	0,69%	
MMV019780	Drug-like	26,55%	10,29%	32,16%	12,11%	2,62%	2,46%	
MMV019871	Drug-like	7,20%	7,42%	12,45%	14,27%	-0,39%	3,25%	
MMV019881	Probe-like	41,52%	4,72%	31,72%	6,69%	16,96%	2,02%	
MMV019995	Probe-like	4,96%	22,25%	14,23%	21,05%	2,53%	0,09%	
MMV020243	Probe-like	-3,73%	9,53%	7,71%	10,16%	1,95%	0,44%	
MMV020275	Drug-like	3,55%	0,23%	9,81%	3,08%	-0,46%	2,77%	
MMV020403	Probe-like	5,86%	7,36%	7,15%	9,43%	4,70%	3,03%	
MMV020439	Drug-like	5,43%	14,37%	15,61%	13,98%	1,47%	0,36%	
MMV020490	Drug-like	-8,63%	5,88%	4,52%	8,49%	0,99%	0,99%	
MMV020492	Drug-like	3,88%	7,56%	9,31%	0,59%	0,71%	0,99%	
MMV020500	Drug-like	45,90%	27,39%	46,13%	16,83%	9,33%	10,51%	
MMV020505	Drug-like	30,93%	6,73%	32,66%	0,10%	7,93%	5,93%	
MMV020548	Drug-like	-2,15%	28,19%	10,04%	24,50%	0,63%	1,34%	
MMV020549	Drug-like	-10,61%	6,36%	0,35%	6,79%	3,76%	1,50%	
MMV020651	Drug-like	11,74%	4,34%	13,09%	1,15%	4,46%	2,44%	
MMV020660	Drug-like	4,01%	6,03%	6,74%	9,62%	3,27%	1,48%	
MMV020700	Drug-like	-1,82%	20,76%	10,43%	19,19%	0,47%	0,40%	
MMV020750	Probe-like	-2,39%	18,21%	8,11%	15,41%	2,82%	2,74%	
MMV020788	Probe-like	58,17%	67,71%	57,76%	55,85%	12,69%	16,08%	unconfirmed hit
MMV020885	Probe-like	19,55%	19,19%	20,69%	16,13%	3,65%	7,38%	
MMV020912	Probe-like	21,76%	14,66%	29,69%	9,12%	1,15%	1,37%	
MMV020942	Drug-like	-16,17%	9,84%	-5,91%	1,73%	-1,85%	0,33%	
MMV056726	Drug-like	-2,41%	2,87%	6,63%	3,40%	-2,76%	0,42%	
MMV073843	Probe-like	26,55%	10,29%	31,63%	10,03%	3,67%	0,82%	
MMV075490	Drug-like	0,98%	0,64%	8,21%	4,55%	-1,24%	0,31%	
MMV080034	Probe-like	28,12%	34,64%	32,91%	29,40%	3,71%	2,45%	

ID	Set	gamete formaton inhibition	± SD	total sexual forms inhibition	± SD	% rounding up inhibition	± SD	comments
MMV084434	Probe-like	4,77%	16,96%	11,46%	6,41%	-0,39%	4,04%	
MMV084940	Drug-like	26,41%	8,08%	29,44%	6,46%	6,85%	2,02%	
MMV085203	Probe-like	28,77%	6,64%	34,63%	7,91%	1,88%	0,91%	
MMV085471	Probe-like	4,17%	5,52%	9,80%	8,66%	0,01%	1,71%	
MMV085583	Probe-like	8,36%	4,10%	12,62%	6,27%	1,17%	2,86%	
MMV086103	Probe-like	15,25%	8,70%	16,85%	8,83%	3,52%	4,60%	
MMV128432	Probe-like	-3,56%	12,39%	7,18%	14,26%	2,53%	1,43%	
MMV142383	Drug-like	-5,93%	14,96%	5,87%	12,92%	2,02%	2,43%	
MMV274073	Drug-like	8,35%	11,98%	11,16%	11,51%	2,78%	5,16%	
MMV396594	Probe-like	-3,34%	10,52%	9,30%	11,08%	0,46%	0,15%	
MMV396595	Drug-like	48,24%	42,04%	50,19%	33,68%	4,71%	1,27%	
MMV396632	Drug-like	-3,36%	11,58%	6,46%	11,91%	3,66%	0,58%	
MMV396633	Drug-like	-5,94%	5,48%	1,78%	0,12%	-0,67%	1,03%	
MMV396635	Probe-like	8,82%	4,43%	14,11%	1,38%	0,11%	0,48%	
MMV396652	Probe-like	-8,98%	3,71%	-2,44%	0,87%	0,87%	3,86%	
MMV396663	Probe-like	3,10%	13,28%	14,19%	12,81%	0,91%	0,67%	
MMV396664	Probe-like	-13,41%	2,84%	0,41%	6,36%	1,23%	1,20%	
MMV396665	Probe-like	0,92%	4,77%	6,15%	0,09%	1,20%	1,50%	
MMV396669	Drug-like	13,93%	18,28%	22,11%	12,15%	2,77%	1,14%	
MMV396672	Drug-like	-4,74%	22,56%	7,27%	22,25%	1,16%	1,19%	
MMV396679	Probe-like	46,73%	16,06%	48,01%	15,45%	4,47%	0,83%	
MMV396680	Probe-like	-15,91%	3,03%	1,04%	4,58%	-1,58%	0,79%	
MMV396681	Drug-like	-1,54%	9,78%	9,53%	10,05%	1,96%	0,75%	
MMV396693	Probe-like	1,38%	5,25%	6,37%	10,34%	1,00%	0,16%	
MMV396703	Drug-like	0,15%	11,00%	6,61%	4,72%	0,15%	0,54%	
MMV396704	Drug-like	-3,87%	20,05%	8,87%	17,33%	0,48%	1,94%	
MMV396705	Drug-like	15,43%	6,75%	17,06%	9,13%	3,40%	2,05%	
MMV396715	Drug-like	43,24%	7,84%	45,39%	8,23%	4,16%	0,19%	
MMV396717	Probe-like	0,34%	13,31%	12,33%	14,02%	0,31%	0,78%	
MMV396719	Drug-like	38,77%	16,01%	39,92%	11,39%	7,64%	1,48%	
MMV396723	Probe-like	41,08%	7,93%	40,71%	1,57%	9,40%	6,15%	
MMV396726	Probe-like	-7,00%	21,12%	0,93%	11,20%	-0,11%	1,59%	
MMV396736	Drug-like	19,85%	0,17%	26,19%	1,49%	3,55%	1,03%	
MMV396744	Drug-like	13,70%	6,80%	21,81%	7,85%	2,39%	0,04%	
MMV396770	Probe-like	3,13%	6,62%	14,26%	3,00%	1,11%	0,04%	
MMV403679	Drug-like	19,06%	12,63%	23,28%	14,61%	6,13%	2,04%	
MMV498479	Probe-like	-7,32%	18,47%	5,68%	21,85%	0,25%	4,60%	
MMV638723	Probe-like	9,13%	4,27%	18,21%	0,59%	2,31%	2,26%	
MMV645672	Probe-like	0,63%	5,17%	13,89%	6,35%	-1,11%	0,37%	
MMV665783	Probe-like	16,36%	9,19%	18,81%	2,09%	2,90%	2,18%	
MMV665785	Probe-like	8,66%	8,74%	10,01%	8,17%	4,47%	5,70%	
MMV665786	Probe-like	-10,81%	11,55%	1,33%	13,35%	2,38%	0,95%	
MMV665789	Drug-like	19,28%	17,07%	28,26%	11,14%	0,31%	1,07%	
MMV665794	Probe-like	26,25%	1,81%	25,33%	2,01%	5,89%	0,35%	

ID	Set	gamete formaton inhibition	± SD	total sexual forms inhibition	± SD	% rounding up inhibition	± SD	comments
MMV665796	Drug-like	13,04%	16,04%	14,14%	18,84%	3,85%	0,86%	
MMV665797	Probe-like	3,85%	4,48%	14,27%	7,40%	1,58%	1,51%	
MMV665798	Drug-like	5,73%	14,67%	12,05%	8,84%	-0,32%	1,16%	
MMV665800	Drug-like	1,92%	2,84%	10,87%	7,10%	3,70%	2,48%	
MMV665803	Drug-like	12,59%	26,69%	18,71%	23,54%	5,10%	1,89%	
MMV665805	Drug-like	49,55%	29,45%	47,85%	19,25%	12,45%	11,02%	
MMV665806	Drug-like	8,97%	6,02%	19,25%	7,24%	0,65%	0,01%	
MMV665807	Drug-like	-11,73%	21,19%	-0,42%	11,08%	-3,11%	1,14%	
MMV665809	Probe-like	3,24%	4,05%	14,45%	3,80%	0,88%	2,44%	
MMV665810	Probe-like	4,54%	11,19%	8,91%	10,52%	1,44%	5,49%	
MMV665812	Probe-like	-9,40%	15,74%	4,00%	13,45%	0,93%	2,46%	
MMV665813	Probe-like	-11,05%	19,11%	2,80%	17,27%	0,67%	1,28%	
MMV665814	Probe-like	37,26%	8,10%	32,04%	1,72%	11,40%	3,94%	
MMV665817	Drug-like	-6,67%	10,85%	3,59%	11,20%	3,77%	0,76%	
MMV665820	Drug-like	-12,29%	7,71%	2,56%	11,08%	-0,25%	2,19%	
MMV665824	Probe-like	3,41%	18,52%	13,63%	15,02%	2,05%	3,39%	
MMV665826	Drug-like	32,39%	12,56%	28,51%	12,42%	9,07%	4,49%	
MMV665827	Probe-like	-1,87%	4,06%	11,26%	5,48%	-0,34%	0,44%	
MMV665831	Probe-like	-9,14%	16,98%	1,64%	9,42%	-3,15%	0,82%	
MMV665836	Probe-like	-7,05%	4,53%	2,48%	9,67%	-2,87%	0,08%	
MMV665840	Probe-like	14,74%	5,85%	21,19%	6,97%	4,36%	0,29%	
MMV665841	Probe-like	22,93%	14,98%	26,10%	13,05%	0,44%	5,89%	
MMV665843	Drug-like	11,17%	4,97%	20,71%	7,47%	1,04%	1,48%	
MMV665850	Drug-like	26,67%	10,77%	33,18%	4,52%	1,96%	3,86%	
MMV665852	Probe-like	16,57%	2,94%	22,45%	3,46%	4,80%	4,13%	
MMV665857	Drug-like	12,79%	30,26%	20,26%	25,06%	3,55%	3,39%	
MMV665864	Probe-like	30,84%	29,50%	20,81%	15,80%	23,59%	17,75%	
MMV665874	Drug-like	-3,97%	9,11%	3,95%	0,55%	-1,01%	1,89%	
MMV665875	Probe-like	27,15%	4,79%	27,56%	7,80%	3,71%	0,53%	
MMV665876	Drug-like	0,56%	5,10%	4,52%	3,16%	2,58%	4,51%	
MMV665879	Drug-like	2,65%	10,03%	8,64%	4,80%	0,31%	1,43%	
MMV665881	Probe-like	-2,49%	0,33%	3,13%	2,19%	1,03%	4,18%	
MMV665882	Probe-like	18,15%	0,27%	25,98%	3,20%	1,83%	1,84%	
MMV665883	Drug-like	38,14%	20,99%	41,54%	17,01%	3,61%	3,87%	
MMV665886	Probe-like	14,66%	17,96%	17,39%	17,22%	1,99%	4,48%	
MMV665888	Drug-like	4,71%	11,79%	8,06%	12,11%	2,51%	4,39%	
MMV665890	Drug-like	26,06%	17,12%	29,16%	14,89%	6,79%	2,86%	
MMV665891	Drug-like	50,61%	64,39%	48,10%	56,30%	8,40%	13,85%	unconfirmed hit
MMV665894	Probe-like	12,12%	7,21%	14,24%	5,76%	3,29%	6,74%	
MMV665897	Drug-like	-6,18%	13,51%	6,05%	12,50%	1,56%	1,51%	
MMV665898	Probe-like	25,40%	10,93%	24,82%	9,69%	5,29%	5,91%	
MMV665899	Drug-like	-9,52%	2,12%	-2,16%	8,08%	-0,14%	0,01%	
MMV665901	Drug-like	12,47%	4,36%	20,80%	3,95%	2,48%	3,38%	
MMV665902	Drug-like	13,64%	14,33%	18,14%	7,70%	0,89%	0,62%	

ID	Set	gamete formaton inhibition	± SD	total sexual forms inhibition	± SD	% rounding up inhibition	± SD	comments
MMV665904	Drug-like	21,30%	11,38%	21,86%	9,70%	4,22%	6,46%	
MMV665906	Drug-like	1,40%	12,84%	11,58%	12,79%	2,35%	0,49%	
MMV665908	Probe-like	15,64%	14,77%	24,15%	9,93%	1,79%	0,15%	
MMV665909	Drug-like	57,89%	2,42%	45,75%	0,22%	22,25%	0,50%	unconfirmed hit
MMV665914	Drug-like	0,20%	0,62%	11,10%	3,24%	1,89%	0,19%	
MMV665915	Drug-like	16,50%	1,29%	26,13%	1,57%	-0,24%	0,73%	
MMV665916	Drug-like	54,90%	72,66%	50,39%	65,59%	17,98%	22,98%	unconfirmed hit
MMV665917	Drug-like	-1,02%	11,38%	10,93%	10,36%	0,85%	1,81%	
MMV665918	Drug-like	9,94%	9,45%	12,40%	10,16%	3,01%	4,03%	
MMV665923	Probe-like	35,42%	23,15%	37,87%	16,62%	6,62%	2,61%	
MMV665924	Probe-like	18,54%	10,79%	18,48%	7,72%	5,31%	8,33%	
MMV665927	Probe-like	-7,97%	9,08%	1,12%	2,52%	-1,75%	0,62%	
MMV665928	Drug-like	0,42%	10,36%	10,14%	10,25%	3,14%	1,16%	
MMV665929	Drug-like	-3,90%	3,59%	8,93%	4,19%	0,48%	1,59%	
MMV665934	Probe-like	12,15%	20,60%	20,46%	16,65%	2,61%	3,60%	
MMV665935	Drug-like	29,25%	11,07%	34,78%	6,95%	2,72%	0,69%	
MMV665936	Probe-like	-0,65%	14,97%	9,72%	13,67%	2,59%	1,61%	
MMV665939	Drug-like	3,89%	6,45%	9,94%	0,31%	-0,06%	0,90%	
MMV665940	Drug-like	-7,38%	17,28%	6,50%	17,33%	-0,28%	0,70%	
MMV665943	Probe-like	4,07%	6,75%	14,12%	9,02%	1,95%	1,17%	
MMV665944	Probe-like	19,26%	1,52%	27,64%	4,21%	0,74%	1,31%	
MMV665946	Drug-like	-8,28%	13,38%	4,55%	13,56%	1,22%	0,23%	
MMV665948	Drug-like	3,50%	18,65%	14,51%	16,60%	0,92%	1,41%	
MMV665949	Probe-like	16,15%	1,52%	14,60%	0,68%	7,28%	6,38%	
MMV665953	Drug-like	1,41%	2,78%	10,12%	6,38%	4,10%	1,65%	
MMV665954	Drug-like	7,49%	9,40%	16,44%	9,80%	2,63%	0,52%	
MMV665961	Drug-like	31,60%	2,27%	33,02%	0,06%	8,18%	0,09%	
MMV665971	Probe-like	-1,50%	4,57%	11,30%	7,71%	-0,13%	1,73%	
MMV665972	Probe-like	-0,96%	15,41%	9,26%	15,37%	2,69%	0,10%	
MMV665977	Probe-like	11,30%	13,04%	19,01%	10,58%	3,53%	3,39%	
MMV665979	Drug-like	10,81%	7,53%	16,41%	8,08%	6,14%	0,95%	
MMV665987	Probe-like	3,25%	7,13%	13,84%	7,12%	1,59%	1,50%	
MMV665994	Probe-like	3,47%	8,20%	10,19%	11,70%	-1,31%	0,82%	
MMV666009	Drug-like	-4,16%	16,58%	7,41%	17,27%	1,66%	1,04%	
MMV666020	Probe-like	6,54%	4,55%	16,09%	4,12%	2,20%	2,56%	
MMV666021	Probe-like	11,22%	17,53%	20,35%	13,98%	1,74%	3,62%	
MMV666022	Probe-like	8,40%	3,46%	16,13%	2,83%	4,07%	3,04%	
MMV666023	Probe-like	56,53%	13,71%	42,48%	16,67%	24,92%	4,60%	
MMV666025	Probe-like	-5,35%	5,74%	0,76%	1,17%	0,97%	0,35%	
MMV666026	Probe-like	28,37%	14,03%	27,22%	10,28%	5,89%	9,00%	
MMV666054	Probe-like	12,63%	11,41%	18,09%	10,29%	6,02%	2,27%	
MMV666057	Drug-like	27,38%	10,22%	32,22%	7,04%	4,14%	0,48%	
MMV666060	Probe-like	-3,27%	10,17%	9,28%	13,22%	0,35%	2,70%	
MMV666061	Drug-like	4,94%	0,10%	14,77%	1,75%	2,21%	0,96%	

ID	Set	gamete formaton inhibition	± SD	total sexual forms inhibition	± SD	% rounding up inhibition	± SD	comments
MMV666062	Probe-like	-5,35%	6,14%	9,34%	9,62%	-1,57%	2,46%	
MMV666067	Drug-like	2,95%	0,62%	8,57%	8,01%	0,14%	3,70%	
MMV666069	Drug-like	8,84%	2,33%	18,30%	5,06%	1,77%	1,01%	
MMV666070	Drug-like	18,71%	21,17%	25,38%	15,72%	3,70%	0,06%	
MMV666071	Drug-like	16,71%	11,05%	24,80%	11,40%	1,64%	0,19%	
MMV666072	Drug-like	-3,86%	4,30%	6,43%	3,49%	3,36%	3,10%	
MMV666075	Drug-like	4,73%	1,36%	11,28%	7,17%	-1,11%	1,02%	
MMV666079	Probe-like	25,43%	15,49%	29,83%	14,17%	4,89%	1,42%	
MMV666080	Drug-like	14,75%	1,13%	22,79%	1,88%	2,37%	0,82%	
MMV666081	Drug-like	20,19%	20,83%	21,94%	19,14%	2,58%	5,22%	
MMV666093	Drug-like	7,26%	14,11%	15,92%	14,24%	2,93%	0,07%	
MMV666095	Probe-like	-2,51%	27,67%	9,55%	24,48%	0,81%	0,91%	
MMV666101	Probe-like	1,57%	3,13%	12,51%	1,06%	1,61%	1,34%	
MMV666102	Drug-like	13,52%	10,92%	22,99%	12,36%	0,41%	1,71%	
MMV666103	Drug-like	92,32%	18,12%	86,46%	15,44%	7,35%	3,42%	unconfirmed hit
MMV666105	Drug-like	7,17%	18,36%	14,65%	17,46%	4,38%	0,63%	
MMV666106	Probe-like	-4,18%	17,65%	7,56%	15,30%	1,73%	2,20%	
MMV666108	Drug-like	3,61%	26,48%	14,41%	24,32%	0,87%	0,21%	
MMV666109	Probe-like	29,07%	18,55%	34,66%	13,14%	2,86%	0,78%	
MMV666110	Drug-like	-1,06%	0,60%	8,45%	1,51%	3,74%	1,83%	
MMV666116	Drug-like	36,66%	30,82%	41,01%	24,06%	3,02%	0,08%	
MMV666123	Probe-like	12,61%	15,24%	15,21%	12,74%	2,60%	7,09%	
MMV666124	Probe-like	8,01%	1,22%	14,34%	4,82%	-1,24%	1,41%	
MMV666125	Probe-like	14,99%	22,28%	16,35%	20,88%	3,61%	5,10%	
MMV666596	Probe-like	-5,87%	23,56%	5,66%	10,73%	-4,05%	4,33%	
MMV666599	Drug-like	5,98%	3,49%	11,02%	1,37%	0,75%	0,78%	
MMV666600	Probe-like	1,56%	1,02%	11,04%	3,15%	3,28%	0,30%	
MMV666601	Probe-like	14,61%	0,68%	21,58%	1,66%	3,82%	0,01%	
MMV666604	Probe-like	-2,96%	5,35%	10,44%	7,17%	-0,47%	0,30%	
MMV666607	Probe-like	45,87%	4,65%	46,61%	0,92%	6,36%	2,97%	
MMV666686	Probe-like	3,22%	23,95%	13,27%	21,88%	1,99%	0,53%	
MMV666687	Probe-like	-2,03%	23,09%	3,81%	16,46%	1,45%	1,61%	
MMV666688	Probe-like	-5,20%	14,45%	8,23%	12,41%	0,02%	2,31%	
MMV666689	Probe-like	14,78%	7,88%	20,06%	4,67%	6,05%	0,21%	
MMV666691	Probe-like	12,22%	2,58%	15,62%	4,61%	1,77%	3,03%	
MMV666692	Probe-like	-1,13%	0,60%	10,12%	2,86%	1,76%	0,88%	
MMV666693	Drug-like	-6,21%	15,77%	1,50%	5,25%	-0,19%	2,95%	
MMV667486	Probe-like	39,80%	25,09%	43,39%	21,29%	2,54%	2,06%	
MMV667487	Drug-like	-3,83%	4,78%	8,44%	7,01%	1,05%	0,51%	
MMV667488	Probe-like	1,44%	8,82%	7,58%	12,21%	-0,36%	1,14%	
MMV667489	Probe-like	-14,91%	7,69%	-5,82%	0,06%	-0,90%	0,19%	
MMV667490	Probe-like	4,73%	8,75%	11,45%	3,01%	-0,86%	0,54%	
MMV667492	Probe-like	5,27%	2,85%	11,60%	2,34%	-0,82%	6,30%	
